# Brain network-driven predictive neural markers for suicide risk

**DOI:** 10.3389/fpsyt.2026.1950068

**Published:** 2026-10-20

**Authors:** Yoojin Lee, Jessica R. Gilbert, Cherise R. Chin Fatt, Jeffrey D. Stout, Laura R. Waldman, Steven J. Lamontagne, Carlos A. Zarate, Elizabeth D. Ballard

**Affiliations:** 1 Experimental Therapeutics and Pathophysiology Branch, National Institute of Mental Health, National Institutes of Health, Bethesda, MD, United States; 2 Center for Depression Research and Clinical Care, Peter O’Donnell Jr. Brain Institute and Department of Psychiatry, University of Texas Southwestern Medical Center, Dallas, TX, United States; 3 Magnetoencephalography Core Facility, National Institute of Mental Health, National Institutes of Health, Bethesda, MD, United States

**Keywords:** default mode network, DIABLO, executive control network, magnetoencephalography, resting-state within-network connectivity, salience network, recent suicidal ideation

## Abstract

**Background:**

Suicide is a significant public health challenge, underscoring the need for temporally precise and objective biomarkers to identify acute suicide risk. One potential biomarker is electrophysiological brain connectivity measured by magnetoencephalography (MEG). This study used publicly available atlases to estimate parcel-level within-network connectivity.

**Methods:**

The multi-omics statistical method was applied to assess whether canonical networks—including the default mode (DMN), executive control (ECN), and salience (SN) networks—could differentiate individuals experiencing a recent suicidal crisis (high risk) from those with a history of suicide attempts but not in the past year (lower risk), clinical controls with depression but no history of suicide risk (clinical controls), and individuals with no personal or family history of either psychiatric disorder or suicidal behavior (minimal risk).

**Results:**

The high risk group exhibited significantly weaker functional connectivity within specific regions of these networks (*ps* <.05). Further analyses revealed that recent suicidal ideation scores were positively associated with functional DMN connectivity (e.g., between the precuneus/posterior cingulate cortex and dorsal/medial prefrontal cortex) and SN connectivity (e.g., between the medial frontal cortex and medial parietal cortex and between the parietal operculum and lateral prefrontal cortex) (*ps* <.05). Conversely, functional ECN connectivity (between the medial/posterior prefrontal cortex and precuneus) was negatively associated with recent suicidal ideation (*ps* <.05).

**Conclusions:**

These findings highlight critical brain regions and network interactions, advancing our understanding of the neural mechanisms underlying suicide risk. Leveraging electrophysiological connectivity and innovative tools may help develop targeted interventions and early identification strategies for those at heightened risk of suicide.

**Clinical trials registration:**

www.clinicaltrials.gov, identifier NCT02543983.

## 1 Introduction

Suicide is a significant public health challenge, accounting for over 700,000 deaths globally each year (1). Understanding the etiology of suicidal behaviors is inherently complex due to our limited mechanistic understanding of suicide risk and to the existence of multiple, distinct pathways that may lead to a suicide attempt. This complexity requires a multifaceted approach to identify reliable, objectively measurable biomarkers of suicide risk. Towards this end, previous research has used a variety of measures, including self-report questionnaires, task-oriented behaviors, and neural markers (2–4). Comprehensive reviews have extensively documented the relationship between suicide risk and both structural and functional neural markers while also highlighting challenges associated with the heterogeneity of how suicide risk is defined (5–7).

Elucidating the temporal dynamics of suicidal thoughts and behaviors is essential for gaining deeper insight into the mechanisms underlying suicide risk and refining its definition. For example, fluctuations in suicidal ideation (SI) over time were found to predict suicide attempts in military populations (8), and the timing of attempts has been associated with impaired cognitive control and neurocognitive function (9). In adolescents with a history of suicide attempts, cognitive performance was found to vary more with the recency of suicidal behaviors than with the specific type of behavior (10, 11). Furthermore, the predictive accuracy of machine learning models for suicide attempts improves when incorporating clinical and demographic data that are temporally proximal to the attempt (12). One study from our laboratory suggested that acute suicidal crises are characterized by downregulated bidirectional glutamatergic feedback between the precuneus and insula, highlighting a potential electrophysiological mechanism underlying acute suicide risk (13). Collectively, these findings indicate that temporally precise data are needed to understand and identify suicide risk as well as mechanistic biomarkers.

Brain connectivity within canonical networks offers a potential way to index mood and suicide risk. In healthy individuals, canonical brain networks measured via the functional connectivity of co-activated brain regions over time—especially the default mode network (DMN), executive control network (ECN), and salience network (SN)—provide critical insights into brain function (14, 15). Menon (16) reviewed the triple network model and demonstrated its relevance to various mental disorders, including mood disorders. Given the high prevalence of suicide in mental disorders (17), particularly depression, the DMN, ECN, and SN may play critical roles in identifying suicide risk. For example, previous studies found that individuals with a history of suicide attempts could be differentiated from those with SI based solely on brain network scores derived from resting-state functional magnetic resonance imaging (fMRI) data, including connectivity in the DMN, SN, ECN, and limbic networks, even without accounting for clinical variables (18). In individuals with bipolar disorder, latent classification of resting-state connectivity using a linear discriminant analysis algorithm revealed that DMN connectivity was positively associated with suicide risk, whereas SN and ECN connectivity were negatively associated (19).

Building on this foundation, multimodal imaging studies identified associations between SI and dysfunctional connectivity in the DMN (20). Suicidal thoughts have also been found to inversely correlate with DMN coherence, reflecting reduced similarity among connections within the DMN (21). The ECN, which supports higher-order cognitive functions such as attention regulation, decision-making, and inhibitory control, has also been implicated in suicide risk (22, 23). Furthermore, behavioral and neuroimaging studies found that individuals at risk for suicide often exhibit impaired executive functioning and reduced cognitive control as well as reduced functional connectivity within fronto-thalamic circuits, which are part of the ECN (24, 25). The SN, which is responsible for detecting emotionally salient stimuli and attention shifting (26), has likewise been associated with suicide risk, with some studies finding negative associations between SN coherence and suicidal thoughts (21, 22, 27).

To further elucidate these relationships, Ordaz and colleagues (22) assessed network coherence by calculating the average normalized correlation between the time series of each voxel within a group-defined network and all other voxels in that network. They found that reduced coherence in the left ECN, anterior DMN, and SN independently correlated with greater lifetime severity of SI; left ECN coherence was the strongest predictor. Recent work using unsupervised learning and connectome-based predictive modeling (28) also found that elevated SI was associated with increased within-network connectivity and decreased external (between-network) connectivity in the DMN, ECN, and dorsal SN, as well as stronger connectivity between the ventral SN and sensorimotor/visual networks (29). These findings suggest that increased within-network connectivity may contribute to suicide risk by promoting maladaptive cognitive processes such as rumination, self-referential thinking, cognitive rigidity, and attentional bias toward negative stimuli (30–33). Given that connections within each network share similar functional properties, within-network connectivity—in contrast to between-network connectivity—may capture functionally specific aspects of suicide risk. However, averaging connectivity across entire networks can obscure important regional heterogeneity within networks, complicating interpretation. Although previous studies identified links between network-level brain connectivity and suicide risk, acute suicidal crises may display distinct neural patterns compared to the chronic suicidal risk state. This study investigated whether specific within-network connectivity patterns in the DMN, ECN, and SN varied according to differing levels of suicide risk.

Magnetoencephalography (MEG) is uniquely suited to assessing brain connectivity using parcel-based orthogonalized envelope correlation, a technique that captures dynamic, phasic changes in electrophysiological signals with much greater temporal resolution than traditional fMRI-based statistical connectivity methods (34, 35). Unlike fMRI, which relies on hemodynamic BOLD signals, MEG directly measures neuronal activity, offering a more immediate reflection of brain function (36). While previous research (18) demonstrated static connectivity differences between suicide attempt and SI groups, MEG could provide temporally precise electrophysiological insights into the neural mechanisms underlying these connectivity patterns. This temporal precision allows detection of dynamic network changes that may be particularly useful for capturing acute suicide risk. MEG also offers superior spatial specificity compared to other electrophysiological techniques such as electroencephalography. Although the full resolution of MEG signals (such as dynamic causal modeling) (37) was not empirically exploited, MEG signals were used to estimate brain connectivity by accounting for temporally precise correlations between two brain regions across time. Defining connectivity at the parcel level using MEG data enhances anatomical interpretability over conventional source-space analyses and enables investigation of how parcel-based connectivity features relate to recent SI scores.

This study used Data Integration Analysis for Biomarker discovery using Latent variable approaches for Omics studies (DIABLO) to assess whether canonical networks—including the DMN, ECN, and SN—could distinguish individuals with a recent suicidal crisis. DIABLO identifies key features across canonical networks and models their relationship, effectively handling high-dimensional, highly correlated datasets. Parcel-level within-network connectivity was estimated using public atlases developed by Schaefer and colleagues (15) to address within-network heterogeneity and to link specific features to recent SI. This approach enabled identification of a concise set of correlated predictors, capturing complex brain network interactions underlying suicide risk.

Here, DIABLO was applied to resting-state MEG data. While an acute suicidal crisis would be expected to reflect a loss of coordinated network function, consistent with reduced coherence reported across canonical networks in suicide risk (20, 21), it is possible that within an acute suicidal crisis, connectivity may instead reflect the intensity of maladaptive processes (30–33). This discrepancy is consistent with evidence that connectivity markers can be either trait-like or state-like (38), especially in the context of a recent suicide attempt (39). Thus, within-network brain connectivity may contain both trait-like and state-like information, reflecting stable vulnerability to suicidal crisis as well as fluctuations in symptom intensity during an active crisis.

The main hypothesis was that individuals with a recent suicidal crisis—either attempted suicide or SI with intent to act within the past two weeks—would exhibit reduced brain connectivity within the DMN—and potentially the ECN and SN as well—due to disrupted internal network organization compared to those with a history of suicide attempts but no current suicidal crisis, clinical controls diagnosed with depression but without suicide risk, and individuals with no personal or family history of either psychiatric disorder or suicidal behavior. Within-network connectivity was used to implement function-based network modeling. This approach serves as a necessary foundation before examining higher-order interactions such as between-network connectivity. It is important to note that previous research investigating within-network connectivity at the individual connection level has been limited, particular in our specific population. Another question of interest was whether, within the suicidal crisis group, higher severity of recent SI would be associated with increased within-network connectivity in the DMN, ECN, and SN.

## 2 Materials and methods

### 2.1 Participants

Data were drawn from the Neurobiology of Suicide protocol, conducted at the National Institute of Mental Health in Bethesda, MD, USA (NCT02543983). Specific inclusion and exclusion criteria have previously been described (40); all participants were either medication-free or maintained on prescribed medications as clinically indicated (see **Table 1**). Eighty participants (19–70 years old) who had at least one resting-state MEG scan were eligible for inclusion in this analysis. Imaging data were low quality for two participants, and one participant had extreme scores for correlation coefficients in the DMN, ECN, and SN (detailed information is provided in the Supplement). As a result, 77 participants (47F; 30M) were included in the study (mean age=38.41 ± 14.09 years). Participants were categorized into four groups based on suicide risk level, determined using the Columbia-Suicide Severity Rating Scale (C-SSRS) and other clinical assessments (41, 42) (see ‘Clinical Ratings’ below): 1) those who had experienced a recent suicidal crisis, defined as either attempted suicide or SI with intent to act within the past two weeks (high risk [HR]; n=8); 2) those with a history of suicide attempt but no suicide attempt or ideation in the past year (lower risk [LR]; n=26); 3) individuals diagnosed with anxiety or mood disorders but with no history of suicide attempt or ideation (clinical controls [CC]; n=22); and 4) individuals with no personal or family history of either psychiatric disorder or suicidal behavior (minimal risk [MR]; n=21) (see **Table 1**). Psychiatric diagnoses were confirmed using the Structured Clinical Interview for DSM Psychiatric Disorders (SCID) (41). Throughout the study, individuals with active suicide risk were managed in the research setting as per our previously published protocol (40). Exclusion criteria included: current drug or alcohol dependence; a severe or unstable medical condition; positive HIV test; pregnancy; a diagnosis of schizophrenia; or not speaking English.

**Table 1:** Demographic information.

Variables	High risk group (HR: n=8)	Lower risk group (LR: n=26)	Clinical controls (CC: n=22)	Minimal risk group (MR: n=21)	Difference statistics
Age	36.78 (9.32)	40.47 (14.78)	40.06 (15.82)	43.69 (12.84)	*F*(3,73) = 0.79, *p* = .502
Sex (Female)	6 (75.00%)	17 (65.38%)	11 (50.00%)	13 (61.90%)	χ^2^(3) = 2.00, *p* = .573
Ethnicity (White)	6 (75.00%)	16 (61.54%)	16 (72.73%)	13 (61.90%)	χ^2^(3) = 1.12, *p* = .772
Medication History (yes)*	7 (87.50%)	14 (53.85%)	19 (86.36%)	–	χ^2^(3) = 37.23, *p <* .001
Poly-Medication (single vs. poly)^&^	4 (57.14%)	5 (35.71%)	9 (68.42%)	–	χ^2^(6) = 41.73, *p <* .001
Primary Diagnosis (MDD)^†^	4(50.00%)	18(69.23%)	22(100.00%)	–	χ^2^(2) = 11.23, *p* = .002
MADRS	30.80 (6.34)	16.30 (9.80)	21.30 (10.90)	0.33 (0.58)	*F*(3,71) = 34.44, *p* < .001
HAM-D24	24.80 (6.58)	14.30 (7.72)	18.80 (9.26)	0.67 (1.06)	*F*(3,71) = 33.87, *p* < .001
HAM-A	16.50 (5.76)	12.40 (6.15)	13.90 (6.91)	0.67 (1.43)	*F*(3,71) = 28.37, *p* < .001
C-SSRS: Recent (<2weeks) Suicidal Ideation^§^	4.75 (0.46)	0.65 (1.06)	0.55 (1.01)	–	*F*(3,73) = 66.94, *p <* .001
C-SSRS: History of Past Suicide Attempts^§^	5 (62.50%)	26 (54.17%)	–	–	χ^2^(3) = 69.20, *p <* .001
C-SSRS: Frequency of Total Suicide Attempts^§^	7.50(13.70)	2.73(2.29)	–	–	*F*(3,73) = 7.05, *p <* .001
SSI: Current Suicidal Ideation^‡^	4.25 (6.41)	1.00 (1.96)	0.73 (1.20)	0.10 (0.45)	*F*(3,70) = 5.80, *p* = .001
SSI: Past Suicidal Ideation^‡^	27.00 (6.53)	26.50 (5.65)	8.42 (8.48)	–	*F*(3,60) = 81.77, *p* < .001
Temporal Delay between Measures (Day)ˆ	1.62 (1.30)	0.65 (1.41)	0.41 (0.50)	0.57 (1.54)	χ^2^(3) = 10.67, *p =* .014

*Any medication prescription history during the study, including selective serotonin reuptake inhibitors (SSRIs), serotonin norepinephrine reuptake inhibitors (SNRIs), or atypical antidepressant drugs: 0 for no and 1 for yes.

^&^ Any poly-drug agents prescribed during the study, including SSRIs, SNRIs, or atypical antidepressant drugs: 0 for no, 1 for single drug-type, and 2 for poly drug-type. The proportion was reported for the poly drug-type.

^†^ Primary diagnoses were reported as major depressive disorder (MDD); other patients were diagnosed with bipolar disorder (BD).

^§^ Recent suicidal ideation (<2weeks), history of past suicide attempts, and frequency of total suicide attempts were measured using the Columbia Suicide Severity Rating Scale (C-SSRS).

^‡^ Both past and current suicidal ideation were measured using the Scale for Suicide Ideation (SSI).

ˆTemporal delay between clinical scale measurements and collection of resting-state magnetoencephalography (MEG) measures.

HAM-A: Hamilton Anxiety Rating Scale; MADRS: Montgomery-Åsberg Depression Rating Scale; HAM-D24: Hamilton Rating Scale for Depression-24; MDD: major depressive disorder.

The Combined Neuroscience Institutional Review Board approved the protocol, and all participants provided written, informed consent. In addition, each patient was assigned a Clinical Research Advocate (CRA) from the Human Subjects Protection Unit (HSPU) who acted as an independent advocate from the initial consent phase through study completion.

### 2.2 Clinical ratings

The Montgomery-Åsberg Depression Rating Scale (MADRS) (43), the Hamilton Rating Scale for Depression 24-item questionnaire (HAM-D24) (44), and the Hamilton Anxiety Rating Scale (HAM-A) (45) were used to assess the severity of psychiatric symptoms. To evaluate both past and current SI, clinicians conducted detailed interviews using the Scale for Suicide Ideation (SSI) (46).

### 2.3 Neuroimaging processes and DIABLO analysis

Resting-state MEG (CTF 275-channel, 1200 Hz, 0–300 Hz bandwidth, synthetic third-order balancing) and T1-weighted MRI scans were collected and co-registered using fiducial markers. MEG data were collected within a day of clinical assessments (M = 0.66 days, SD = 1.27) except for the SR group, which had longer intervals (M = 1.62 days, SD = 1.30) due to last-minute scheduling challenges inherent to the group (see **Table 1**). Resting-state MEG data were processed with MNE python (v1.3) (47), minimum norm estimated and projected (2–58 Hz) onto 400 Schaefer atlas parcels across 17 networks (15), parcels orthogonalized (34), and intra-network envelope correlations (DMN, ECN, SN) computed for analysis. DIABLO (mixOmics, R v6.30.0) (48) was used to integrate 2,628 DMN, 1,225 ECN, and 1,176 SN connectivity features from resting-state MEG, using supervised multi-block sparse partial least square (sPLS) models with a tuned design matrix to identify correlated variables distinguishing participant groups. Detailed information is provided in the Supplement.

### 2.4 Data analysis plan

To identify outliers, brain connectivity scores in the DMN, ECN, and SN were assessed using principal components analysis (PCA) via the epPCA function in R (49). One participant who showed consistently elevated scores across all principal components was identified as an outlier and excluded from this analysis. The remaining standardized connectivity scores were used as model inputs, with group membership (HR, LR, CC, MR) as the outcome.

Two different approaches were used for the DIABLO analysis: 1) the sPLS approach, where inter-network correlations were used as weights in the design matrix, and 2) the discrimination approach, which maximized group separation by entering 0.1 in the design matrix (50–52). Given the high inter-correlations among canonical networks of MEG connectivity features, the discrimination approach—which maximizes group separation by minimizing the influence of shared variance across features—was deemed more appropriate than the sPLS approach, as the latter may preserve the natural correlation structure between networks but could be susceptible to multicollinearity when the design matrix weights reflect highly correlated inter-network relationships. Detailed information is provided in the Supplement.

Brain connectivity scores in each canonical network for the first and second components that most effectively differentiated group membership were exported and used for follow-up analyses. Lasso regression identified the most predictive scores, which were then analyzed by MANCOVA with group membership as the independent variable, brain connectivity scores as dependent variables, and sex, age, and primary diagnosis as covariates. The multiple comparison correction was also applied to the series of MANOVA results and mean differences in brain connectivity scores across canonical networks, with each canonical network treated as a family of comparisons.

To assess clinical utility, linear regression examined associations between connectivity and current SI scores, including sex and age as covariates; only scores with significant group differences were reported. For non-normality and small samples, Bayesian GLMMs (the brms function in R (53)) were used, with MCMC (4 chains, 5000 iterations, 1500 warmup; priors normal). Iterations and warmup were increased if convergence issues arose (R-hat>1.1) (54). Scores predictive in linear models were included in Bayesian analyses, again controlling for sex and age.

## 3 Results

### 3.1 Descriptive statistics

The HR, LR, CC, and MR groups did not differ significantly in terms of age, sex, or ethnicity (*ps*>.05; see **Table 1**; **Supplementary Table 1**). However, mood scale scores (MADRS, HAM-D24, and HAM-A) differed between the groups (*ps* <.05). Detailed group differences are provided in the Supplement. When examining current SI scores, as measured by the SSI, the HR group had significantly higher scores than the LR, CC, and MR groups (LR: MD=-3.25, *p* = .007; CC: MD=-3.52, *p* = .004; MR: MD=-4.15, *p* <.001). For past SI, also measured using the SSI, the HR group had significantly higher scores than the CC (MD=-18.58, *p* <.001) and MR groups (MD=-27.00, *p* <.001), but there was no significant difference between the HR and LR groups (MD=-0.45, *p* = .998).

### 3.2 DIABLO analysis

DIABLO analysis was conducted using both the sPLS and discrimination approaches, but only the discrimination approach significantly differentiated the groups (*p* = .018; sPLA approach: *p* = .319). The inter-component correlation and sample plots demonstrated that network-specific group discrimination was not clearly distinct across networks (**Figures 1A, B**). The arrow plot revealed greater within-group agreement for the LR and CC groups compared to the HR and MR groups (**Figure 1C**). The correlation circle plot indicated predominantly negative correlations between connectivity scores in the first component and positive correlations in the second, with the SN exhibiting higher similarity between connectivity scores than the DMN or ECN (**Figure 1D**). The model then provided the most influential features for group comparisons (**Figure 2**). To identify the minimal set of influential features that could predict group differences, a series of ANCOVA analyses was used to assess whether the selected neural correlates differed between groups, with sex and age included as covariates. The HR group showed reduced within-network connectivity across the DMN, ECN, and SN compared to the other groups, with one exception—there was enhanced connectivity between the lateral prefrontal cortex (lPFC) and parietal operculum (ParOper) versus the CC group. These reductions included: DMN connectivity between the dorsolateral prefrontal cortex (dlPFC) and retrosplenial cortex/medial prefrontal cortex (mPFC) and between the dlPFC and joint area of the precuneus and posterior cingulate cortex (pCun/PCC); ECN connectivity between frontal-parietal regions (including precuneus (pCun)); and SN connectivity between frontal regions (including the medial frontal cortex (mFC) and lPFC) and sensory/emotion-related regions (including the insula and parietal regions). Detailed group differences are provided in the Supplement.

**Figure 1:**
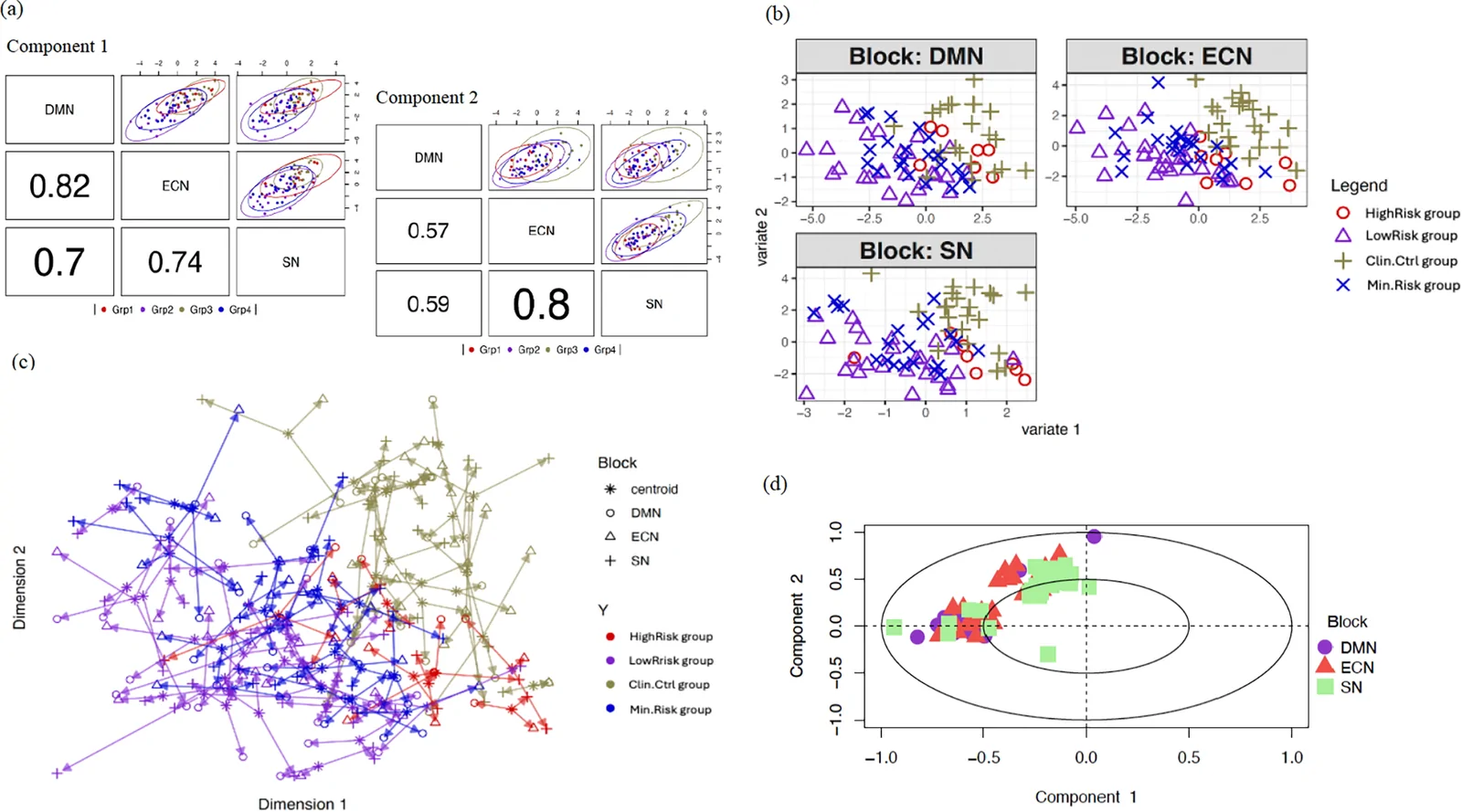
Visualization of Data Integration Analysis for Biomarker discovery using Latent variable approaches for Omics studies (DIABLO) model outcomes. **(a)** Inter-component correlation between canonical networks. **(b)** Sample plot of participant projections onto canonical network component space. **(c)** Arrow plot depicting each participant’s centroid among canonical networks and their specific positions within each network. **(d)** Correlation circle plot depicting inter-network correlations in components. DMN, default mode network; ECN, executive control network; Grp1, high risk; Grp2, lower risk; Grp3, clinical controls; Grp4, minimal risk; SN, salience network.

**Figure 2:**
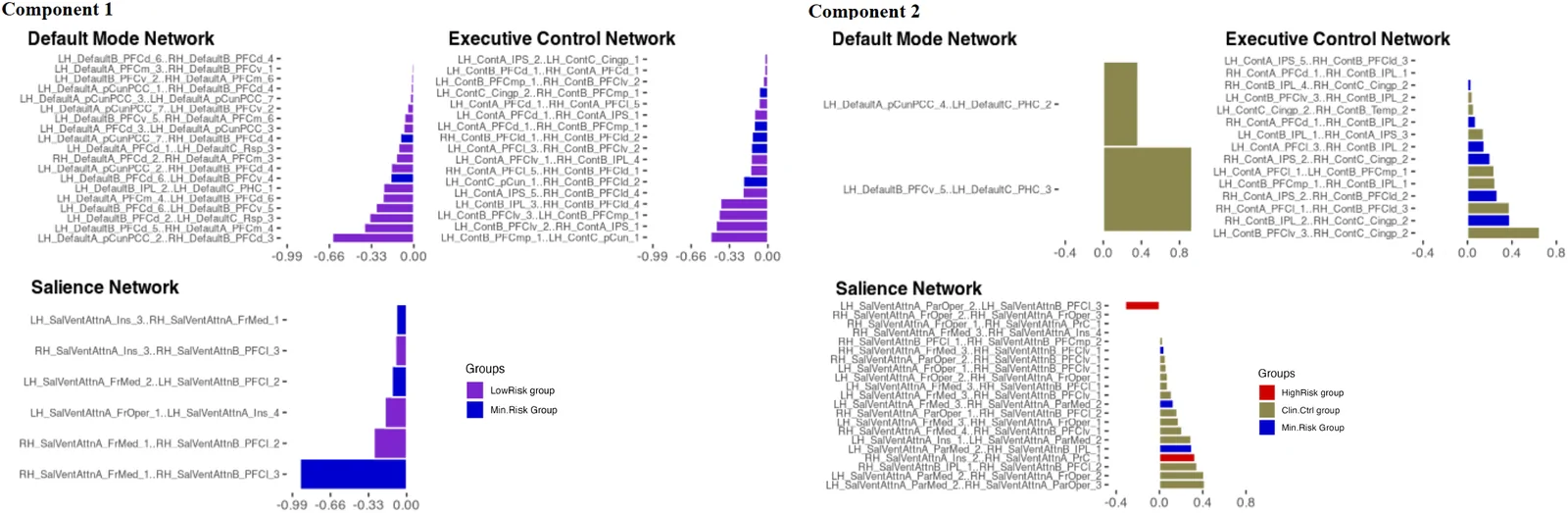
Loadings of each contributing feature within canonical networks for group comparison in the Data Integration Analysis for Biomarker discovery using Latent variable approaches for Omics studies (DIABLO) model. A Ins, anterior insula; CingP, posterior cingulate cortex; ContA, executive control network type A; ContB, executive control network type B; ContC, executive control network type C; DefaultA, default mode network type A; Default B, default mode network type B; Default C, default mode network type C; FrMed, medial frontal cortex; FrOper, frontal operculum; IPL, inferior parietal lobule; IPS, intraparietal sulcus; LH, left hemisphere; ParMed, medial parietal cortex; ParOper, parietal operculum; PCC, posterior cingulate cortex; PCun, precuneus; PCunPCC, precuneus posterior cingulate cortex; PFC, prefrontal cortex; PFCd, dorsal prefrontal cortex; PFCl, lateral prefrontal cortex; PFCld, dorsolateral prefrontal cortex; PFClv, ventrolateral prefrontal cortex; PFCm, medial prefrontal cortex; PFCmp, posterior medial prefrontal cortex; PFCv, ventral prefrontal cortex; PHC, parahippocampal cortex; PrC, precentral cortex; RH, right hemisphere; Rsp, retrosplenial cortex; SalVentAttnA, salience network type A; SalVentAttnB, salience network type B; Temp, temporal cortex.

### 3.3 Specified brain regions and clinical scales

To explore the role of brain correlates in suicide risk between groups, simple linear regression analyses were conducted using the influential neural connectivity features, with recent SI measured via the SSI as the predictor. Differences were observed between the HR group and the three other groups (**Table 2**, **Supplementary Table 5**). Only interaction effects of group on the relationship between the severity of current SI scores and resting-state connectivity in the DMN, ECN, and SN are reported. The relationship between depression severity scores (as measured by the MADRS) and resting-state connectivity from the DMN, ECN, and SN were not moderated by the groups (*ps* <.05). However, compared to the MR group, the HR group displayed a positive interaction between current suicidal thoughts and DMN connectivity, including between the pCun/PCC and dlPFC (β=12.42, se=3.58, *p* = .001) and between the dlPFC and mPFC (β=7.71, se=1.87, *p* <.001) as well as a negative interaction between current suicidal thoughts and DMN connectivity between the pCun/PCC and dlPFC (β=-5.57, se=2.76, *p* = .048; **Figure 3**). In addition, compared to the MR group, the HR group showed a negative interaction between current suicidal thoughts and ECN connectivity between the posterior medial prefrontal cortex (pmPFC) and pCun (β=-5.97, se=2.55, *p* = .022). Compared to the MR group, the HR group showed a positive interaction between current suicidal thoughts and SN connectivity, including between the mFC and mPC (β=8.26, se=2.62, *p* = .002) and between the ParOper and lPFC (β=-5.97, se=2.55, *p* = .022).

**Table 2:** Results of Bayesian analysis of connectivity in canonical networks and recent suicidal ideation.

Reference group	Parameters		Default mode network			Executive control network	Salience network	
			LH : posterior pCun/PCC & RH:dlPFC	LH:pCun/PCC & RH:dlPFC	LH:dlPFC & RH:mPFC	LH:pmPFC & LH:pCun	LH:mFC& RH:mPC	LH : ParOper & LH:lPFC
LR group	Intercept	Estimate	1.72	1.77	2.01	1.91	1.77	1.45
		Est.Error	0.44	0.42	0.22	0.31	0.32	0.33
		Upper	0.76	0.78	1.54	1.18	1.14	0.74
		Lower	3.11	2.50	2.50	2.49	2.52	2.11
		Rhat	1.09	1.01	1.01	1.01	1.02	1.01
		Bulk_ESS	33	555	944	308	202	745
		Tail_ESS	23	367	814	223	66	319
	BC	Estimate	-0.02	0.02	0.16	0.10	-0.01	-0.18
		Est.Error	0.08	0.09	0.08	0.09	0.10	0.10
		Upper	-0.17	-0.15	-0.01	-0.08	-0.20	-0.38
		Lower	0.15	0.20	0.21	0.29	0.19	0.02
		Rhat	1.00	1.00	1.00	1.01	1.01	1.01
		Bulk_ESS	842	873	1034	597	1355	1854
		Tail_ESS	1226	1118	1089	380	1286	2130
	HR group	Estimate	-0.97	2.60	1.43	-1.15	1.74	1.20
		Est.Error	0.64	0.85	0.38	0.56	0.69	0.31
		Upper	-2.26	0.94	0.64	-2.20	0.40	0.60
		Lower	0.28	4.18	2.13	-0.04	3.04	1.82
		Rhat	1.01	1.00	1.00	1.00	1.01	1.00
		Bulk_ESS	1199	1049	969	830	541	1738
		Tail_ESS	2174	1097	840	1870	496	1059
	CC group	Estimate	0.08	0.01	-0.24	-0.46	-0.22	0.24
		Est.Error	0.31	0.34	0.31	0.37	0.27	0.35
		Upper	-0.52	-0.68	-0.83	-1.15	-0.75	-0.48
		Lower	0.67	0.69	0.42	0.29	0.32	0.92
		Rhat	1.00	1.00	1.00	1.01	1.00	1.00
		Bulk_ESS	744	903	937	590	1189	884
		Tail_ESS	1691	827	977	584	1329	232
	MR group	Estimate	-0.19	-0.30	-0.44	-0.35	-0.17	0.13
		Est.Error	0.28	0.29	0.25	0.29	0.28	0.30
		Upper	-0.76	-0.89	-0.94	-0.92	-0.72	-0.46
		Lower	0.35	0.23	0.06	0.23	0.35	0.71
		Rhat	1.01	1.00	1.00	1.01	1.01	1.01
		Bulk_ESS	1093	694	1026	1.66	1327	1631
		Tail_ESS	1007	886	1210	2064	2403	2332
	BC*HR group	Estimate	-0.53	0.98	0.50	-0.68	0.59	0.54
		Est.Error	0.27	0.37	0.18	0.25	0.29	0.18
		Upper	-1.08	0.23	0.12	-1.18	0.03	0.18
		Lower	0.01	1.64	0.84	-0.17	1.12	0.91
		Rhat	1.01	1.00	1.00	1.00	1.01	1.00
		Bulk_ESS	1314	1052	963	1057	464	1652
		Tail_ESS	2111	1084	870	1882	507	1791
	BC*CC group	Estimate	0.06	0.02	-0.15	-0.21	-0.10	0.17
		Est.Error	0.15	0.16	0.15	0.18	0.14	0.16
		Upper	-0.23	-0.30	-0.43	-0.57	-0.38	-0.14
		Lower	0.34	0.34	0.19	0.13	0.17	0.49
		Rhat	1.00	1.00	1.00	1.01	1.00	1.00
		Bulk_ESS	806	876	873	635	1334	1178
		Tail_ESS	1821	787	987	427	1341	744
	BC*MR group	Estimate	-0.03	-0.10	-0.20	-0.13	-0.03	0.15
		Est.Error	0.15	0.16	0.14	0.16	0.15	0.16
		Upper	-0.32	-0.41	-0.47	-0.44	-0.32	-0.15
		Lower	0.25	0.20	0.08	0.18	0.25	0.46
		Rhat	1.01	1.00	1.00	1.01	1.01	1.01
		Bulk_ESS	1000	720	1031	694	1403	1635
		Tail_ESS	1008	929	1193	1735	2807	2332
	Bayes R2		52.75%	56.05%	63.18%	64.08%	56.63%	59.79%
	LOOCV (<.07)		85.50%	92.10%	92.10%	89.50%	100%	92.10%
CC group	Intercept	Estimate	1.76	1.81	1.80	1.46	1.52	1.71
		Est.Error	0.52	0.34	0.40	0.39	0.25	0.39
		Upper	0.74	1.12	1.06	0.65	1.04	0.99
		Lower	2.81	2.53	2.51	2.23	2.05	2.46
		Rhat	1.03	1.01	1.01	1.01	1.01	1/-3
		Bulk_ESS	115	412	673	779	1101	256
		Tail_ESS	153	448	599	779	1351	294
	BC	Estimate	0.03	0.05	0.03	-0.11	-0.10	-0.01
		Est.Error	0.12	0.12	0.13	0.14	0.10	0.12
		Upper	-0.22	-0.19	-0.22	-0.39	-0.30	-0.25
		Lower	0.28	0.31	0.28	0.17	0.09	0.23
		Rhat	1.04	1.00	1.01	1.01	1.00	1.03
		Bulk_ESS	1421	682	573	952	830	425
		Tail_ESS	1786	787	1307	2051	1920	366
	HR group	Estimate	-1.03	2.54	1.65	-0.74	1.94	0.95
		Est.Error	0.67	0.84	0.46	0.61	0.68	0.37
		Upper	-2.35	0.93	0.74	-1.87	0.63	0.23
		Lower	0.36	4.21	2.51	0.44	3.22	1.63
		Rhat	1.01	1.03	1.01	1.01	1.00	1.02
		Bulk_ESS	1503	774	499	978	1234	369
		Tail_ESS	1972	859	1699	2196	959	886
	LR group	Estimate	-0.05	-0.02	0.22	0.43	0.21	-0.26
		Est.Error	0.30	0.33	0.32	0.35	0.27	0.34
		Upper	-0.65	-0.73	-0.41	-0.24	-0.31	-0.92
		Lower	0.56	0.52	0.81	1.11	0.77	0.40
		Rhat	1.02	1.01	1	1.01	1.00	1.01
		Bulk_ESS	1418	243	646	577	1482	675
		Tail_ESS	2013	791	1053	1672	2134	836
	MR group	Estimate	-0.25	-0.32	-0.25	0.08	0.03	-0.14
		Est.Error	0.35	0.35	0.35	0.37	0.27	0.37
		Upper	-0.95	-1.03	-0.91	-0.67	-0.49	-0.83
		Lower	0.44	0.35	0.47	0.84	0.55	0.59
		Rhat	1.04	1.00	1.01	1.04	1.01	1.01
		Bulk_ESS	1722	852	1129	1608	1364	717
		Tail_ESS	2237	1055	1418	2297	321	992
	BC*HR group	Estimate	-0.58	0.94	0.63	-0.48	0.68	0.37
		Est.Error	0.28	0.36	0.22	0.27	0.28	0.19
		Upper	-1.14	0.24	0.22	-1.01	0.14	0.01
		Lower	0.01	1.64	1.04	0.03	1.25	0.72
		Rhat	1.01	1.02	1.01	1.01	1.00	1.02
		Bulk_ESS	1551	801	426	1123	1080	398
		Tail_ESS	1861	825	555	2300	1088	861
	BC*LR group	Estimate	-0.04	-0.02	0.13	0.21	0.10	-0.17
		Est.Error	0.15	0.16	0.16	0.17	0.14	0.16
		Upper	-0.32	-0.35	-0.17	-0.12	-0.17	-0.48
		Lower	0.26	0.25	0.41	0.53	0.39	0.13
		Rhat	1.02	1.01	1.00	1.01	1.00	1.01
		Bulk_ESS	1400	258	588	659	941	644
		Tail_ESS	2184	866	694	1854	2250	633
	BC*MR group	Estimate	-0.08	-0.12	-0.08	0.07	0.07	-0.03
		Est.Error	0.17	0.18	0.17	0.18	0.15	0.17
		Upper	-0.42	-0.48	-0.40	-0.29	-0.21	-0.36
		Lower	0.26	0.22	0.27	0.43	0.35	0.34
		Rhat	1.03	1.01	1.01	1.03	1.01	1.00
		Bulk_ESS	1750	891	1256	1486	1291	655
		Tail_ESS	1979	1091	1708	2082	1750	993
	Bayes R2		54.01%	55.60%	67.93%	61.19%	59.13%	61.71%
	LOOCV (<.07)		90.80%	89.50%	89.50%	94.70%	96.10%	96.10%
MR group	Intercept	Estimate	1.54	1.52	1.57	1.54	1.56	1.57
		Est.Error	0.31	0.40	0.29	0.32	0.29	0.35
		Upper	0.93	0.66	1.01	0.95	0.90	0.81
		Lower	2.14	2.22	2.14	2.19	2.13	2.20
		Rhat	1.01	1.01	1.03	1.01	1.02	1.01
		Bulk_ESS	396	394	222	557	539	413
		Tail_ESS	714	377	257	1205	167	797
	BC	Estimate	-0.05	-0.06	-0.03	-0.04	-0.04	-0.03
		Est.Error	0.11	0.13	0.12	0.13	0.10	0.13
		Upper	-0.27	-0.32	-0.24	-0.29	-0.23	-0.29
		Lower	0.17	0.22	0.20	0.20	0.17	0.23
		Rhat	1.01	1.01	1.02	1.00	1.00	1.01
		Bulk_ESS	747	804	442	1267	786	291
		Tail_ESS	1017	839	330	1299	995	998
	HR group	Estimate	-0.84	2.84	1.82	-0.76	1.95	1.08
		Est.Error	0.69	0.83	0.42	0.57	0.70	0.36
		Upper	-2.20	1.20	0.95	-1.87	0.56	0.33
		Lower	0.48	4.46	2.59	0.37	3.25	1.73
		Rhat	1.01	1.00	1.02	1.01	1.02	1.00
		Bulk_ESS	426	892	411	1169	271	518
		Tail_ESS	945	965	304	1517	1017	1004
	LR group	Estimate	0.21	0.25	0.44	0.36	0.18	-0.13
		Est.Error	0.27	0.29	0.26	0.29	0.27	0.31
		Upper	-0.03	-0.34	-0.08	-0.17	-0.37	-0.73
		Lower	0.71	0.79	0.95	0.96	0.69	0.50
		Rhat	1.03	1.01	1.01	1.00	1.01	1.01
		Bulk_ESS	392	916	498	1028	794	514
		Tail_ESS	701	985	729	1363	735	1022
	CC group	Estimate	0.25	0.28	0.19	-0.05	-0.05	0.13
		Est.Error	0.36	0.36	0.38	0.39	0.29	0.37
		Upper	-0.46	-0.45	-0.54	-0.82	-0.59	-0.56
		Lower	0.97	0.97	0.91	0.71	0.55	0.88
		Rhat	1.02	1.01	1.02	1.01	1.02	1.00
		Bulk_ESS	382	618	595	1471	203	693
		Tail_ESS	161	922	479	1527	381	966
	BC*HR group	Estimate	-0.53	1.04	0.67	-0.53	0.63	0.39
		Est.Error	0.29	0.36	0.21	0.26	0.29	0.19
		Upper	-1.10	0.32	0.25	-1.04	0.06	0.01
		Lower	0.05	1.75	1.05	-0.02	1.16	0.74
		Rhat	1.01	1.01	1.02	1.00	1.02	1.01
		Bulk_ESS	462	875	411	1281	337	589
		Tail_ESS	1017	883	593	1333	832	1011
	BC*LR group	Estimate	0.04	0.07	0.19	0.15	0.03	-0.15
		Est.Error	0.14	0.16	0.14	0.16	0.14	0.17
		Upper	-0.25	-0.25	-0.08	-0.17	-0.26	-0.47
		Lower	0.30	0.38	0.48	0.47	0.29	0.17
		Rhat	1.02	1.01	1.01	1.00	1.01	1.01
		Bulk_ESS	552	885	461	1271	835	395
		Tail_ESS	763	857	506	1333	1199	1053
	BC*CC group	Estimate	0.08	0.09	0.05	-0.06	-0.08	0.02
		Est.Error	0.17	0.18	0.18	0.19	0.16	0.17
		Upper	-0.24	-0.26	-0.29	-0.43	-0.36	-0.31
		Lower	0.42	0.44	0.40	0.31	0.24	0.37
		Rhat	1.02	1.00	1.02	1.01	1.03	1.00
		Bulk_ESS	395	585	548	1432	106	659
		Tail_ESS	468	937	551	1258	429	939
	Bayes R2		57.15%	57.93%	62.01%	59.02%	54.75%	60.62%
	LOOCV (<.07)		90.80%	97.40%	88.20%	88.20%	89.50%	90.80%

BC, brain connectivity; HR, high risk group; LR, lower risk group; CC, clinical controls; MR, minimal risk group; ESS, effective sample size; LOOCV, leave-one-out cross-validation; RH, right hemisphere; LH, left hemisphere; pCun/PCC, precuneus posterior cingulate cortex; dlPFC, dorsolateral prefrontal cortex; mPFC, medial prefrontal cortex; pmPFC, posterior medial prefrontal cortex; mPC, medial parietal cortex; mFC, medial frontal cortex; ParOper, parietal operculum; lPFC, lateral prefrontal cortex.

Based on the ESS measures, including central and tails of the posterior distribution, the warmup period was increased to reduce the influence of initial values. If the Rhat score indicated a large value (>1.1), suggesting non-convergence of the same posterior distribution in different Markov Chain Monte Carlo (MCMC) chains, both the number of iterations and the warmup period were increased.

**Figure 3:**
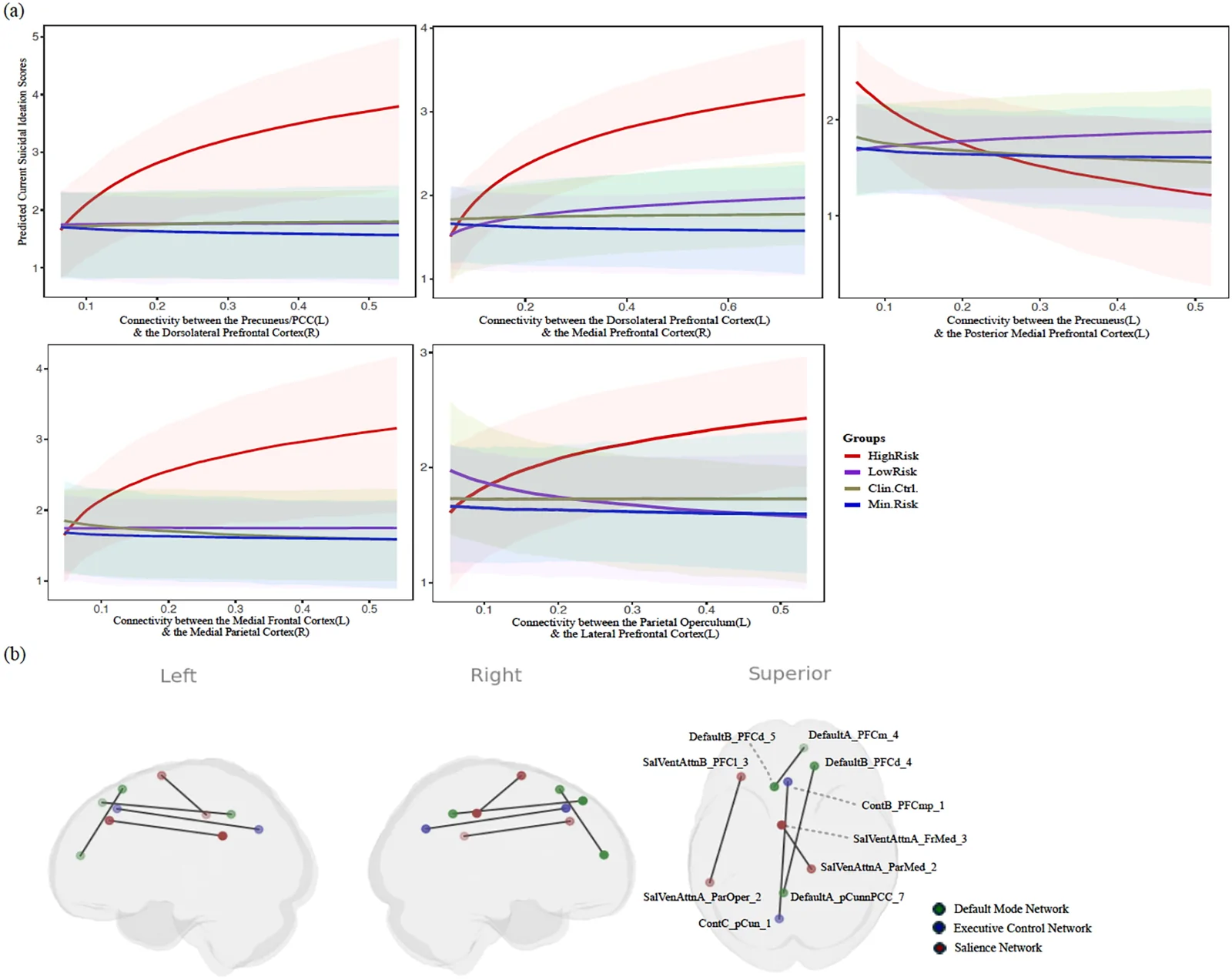
Relationship between connectivity in canonical networks and recent suicidal ideation (SI). **(a)** Results of the Bayesian regression of connectivity in predicting recent SI. **(b)** Mapping the location of connectivity in canonical networks. ContB, executive control network type B; ContC, executive control network type C; DefaultA, default mode network type A; Default B, default mode network type B; Default C, default mode network type C; FrMed, medial prefrontal cortex; ParMed, medial parietal cortex; ParOper, parietal operculum; Pcun, precuneus; PCunPCC, precuneus posterior cingulate cortex; PFCd, dorsal prefrontal cortex; PFCl, lateral prefrontal cortex; PFCm, medial prefrontal cortex; PFCmp, posterior medial prefrontal cortex; SalVentAttnA, salience network type A; SalVentAttnB, salience network type B.

To tackle non-normality and limited sample size concerns, a Bayesian linear mixed model (estimated using MCMC sampling with four chains of 5000 iterations and a warmup of 1500) was fitted to predict the severity of current SI (**Table 2**, **Figure 4**). If the model showed low reliability (<400) based on Bulk_ESS and Tail_ESS scores with four individual chains (54), the warmup period was increased from 1500 to 3500 to minimize the effects of initial values. For one model that did not converge, iteration numbers were increased from 5000 to 15,000. Overall, the models successfully converged (Rhat<1.1) with reasonable reliability. Resting-state DMN connectivity between the pCun/PCC and dlPFC, as well as between the dlPFC and mPFC, positively predicted the severity of current SI in the HR group compared to the LR, CC, and MR groups. However, resting-state DMN connectivity between the posterior pCun/PCC and dlPFC did not predict the severity of current SI in the HR group compared to the LR, CC, and MR groups. Resting-state ECN connectivity between the pCun and pmPFC negatively predicted the severity of current SI in the HR group compared to the LR and MR groups. Lastly, resting-state SN connectivity between the mFC and mPC, as well as between the ParOper and lPFC, positively predicted the severity of current SI in the HR group compared to the LR, CC, and MR groups. Results of the sensitivity analyses are provided in the Supplement.

**Figure 4:**
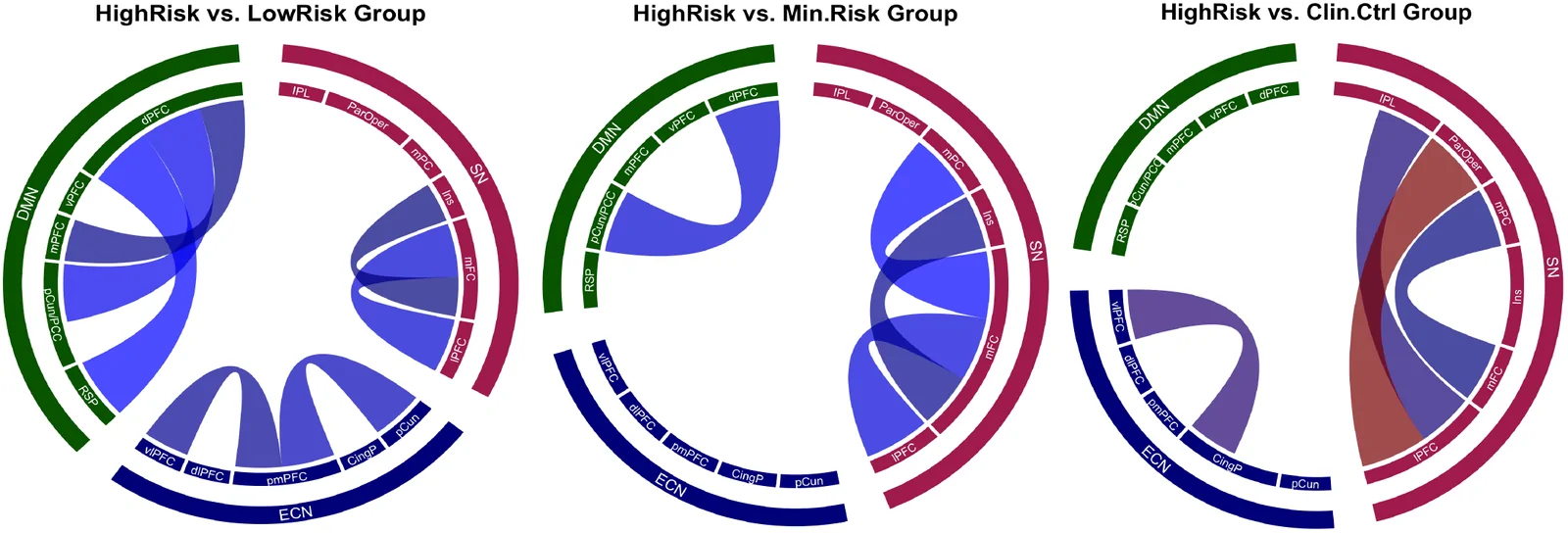
Circos plot of selected internal connectivity within canonical networks. DMN, default mode network; ECN, executive control network; SN, salience network; dPFC, dorsal prefrontal cortex; vPFC, ventral prefrontal cortex; mPFC, medial prefrontal cortex; pCun/PCC, precuneus posterior cingulate cortex; RSP, retrosplenial cortex; vlPFC, ventrolateral prefrontal cortex; dlPFC, dorsolateral prefrontal cortex; pmPFC, posterior medial prefrontal cortex; CingP, cingulate posterior; pCun, precuneus; lPFC, lateral prefrontal cortex; mFC, medial frontal cortex; Ins, insula; mPC, medial parietal cortex; ParOper, parietal operculum; IPL, inferior parietal lobule.

## 4 Discussion

This study used a DIABLO-based analytic approach to investigate whether MEG-driven intra-network brain connectivity could differentiate individuals experiencing a recent suicidal crisis from those with a history of suicide attempts, clinical controls with depression but no suicide risk, and individuals with no personal or family history of either psychiatric disorder or suicidal behavior. The working hypothesis was that individuals who had experienced a recent suicidal crisis would show reduced brain connectivity within the DMN, ECN, and SN compared to other groups. Indeed, the results showed that the HR group had weaker functional brain connectivity within specific brain regions of these networks, partially supporting our hypothesis. Further analyses revealed positive associations between recent SI scores (measured by the SSI) and functional DMN connectivity (between the pCun/PCC and dlPFC and between the dlPFC and mPFC) as well as functional SN connectivity (between the mFC and pFC as well as between the ParOper and lPFC). Conversely, functional ECN connectivity (between the pCun and pmPFC) was negatively associated with SI scores. These findings highlight key brain regions and differences in connectivity that enhance our understanding of the neural mechanisms underlying suicide risk.

This study used a novel approach to investigate the utility of brain connectivity in the three canonical networks of interest for predicting group membership in different suicide risk groups (HR, LR, CC, and MR). Applying DIABLO—which was originally developed to examine -omics data (55) and has recently been applied to resting-state fMRI data (56)—to MEG brain connectivity scores accounted for intra-network relationships and helped assess the contribution of individual connectivity features to group classification. Importantly, traditional dimension-reduction or feature-selection methods, such as independent component analysis, lasso regression, or k-means clustering, cannot account for the multi-cluster structure of inputs (i.e., different within-network connectivity scores) and often present challenges in functional interpretation (19, 57, 58). Furthermore, relationships between multi-cluster inputs cannot be investigated using these traditional methods. In contrast, DIABLO—which is characterized by integrative supervision and a parcel-based envelope correlation approach—enabled identification of key MEG connectivity features while accounting for the multi-cluster structure of inputs and preserving interpretability via feature selection. This method not only enhanced classification accuracy but also provided novel insights into the neural mechanisms underlying the suicidal crisis by clarifying connectivity within canonical networks. Furthermore, although the relationship between brain connectivity and suicide risk has been widely examined using resting-state fMRI data (18, 59), MEG-based studies remain scarce. Our application of DIABLO to electrophysiological data represents an innovative advance in multi-modal brain research, with the potential to identify robust biomarkers of suicide risk and related psychiatric conditions. It is worth noting that, compared to the other groups, the HR group comprised individuals with greater severity of depressive and anxiety symptoms as well as a more extensive medication history. Sensitivity analyses showed that the relationships between selected MEG connectivity features and recent SI were attenuated when severity of depressive and anxiety symptoms and medication history were included as covariates. It remains unclear whether the identified MEG connectivity features within the canonical networks reflect the recent suicidal crisis or are partially attributable to severe depression, anxiety, and medication use.

Taken together, the results suggest possible dysfunction within the DMN, ECN, and SN in individuals who experienced a recent suicidal crisis. Moreover, specific subsets of these networks were associated with recent suicidal thoughts, supporting previous findings of DMN dysfunction in individuals with mood disorders (20). Several brain regions have been implicated in the manifestation of SI, including the dlPFC, the inferior frontal gyrus (IFG), and the PCC. The dlPFC is responsible for processing emotional responses to negatively-valenced social cues, which are mediated by risky or temporally discounted decision-making in suicide attempts (4, 60). The IFG is associated with cognitive control and exhibits increased activity in response to social stimuli, which are linked to impulsivity (61, 62). Finally, the PCC plays a role in cognitive control, especially in response to negative social cues, and is involved in self-referential processes (32, 63).

This study found increased brain connectivity within the DMN, specifically between the dlPFC and pCun/PCC and between the dlPFC and mPFC. The mPFC is involved in cognitive and emotional processes and their integration (64, 65). In the context of suicide, the dlPFC may play a role in executive functions such as spatial planning and problem solving (66). Furthermore, multiple studies have identified an association between the PCC and suicide risk, including variability of its connectivity to the IFG in individuals with suicidal thoughts (67) and a negative relationship between suicidal thoughts and brain connectivity between the PCC and cerebellum, lateral occipital gyrus, and temporal-occipital fusiform gyrus (68).

Although the mPFC has been less explored in relation to suicide risk, it is known to be associated with updating and mediating social dominance status (69) and retrieving negative emotional experiences (70). Our findings of a positive relationship between suicidal thoughts and select DMN connectivity involving the dlPFC, PCC, and mPFC suggest an amplification of cognitive and emotional processes and the integration of negative social cues, particularly those related to death. For instance, a previous study found increased connectivity from the orbital frontal cortex to the PCC in response to death-related visual stimuli (71). Moreover, recent research indicates that SI is associated with enhanced connectivity between the dlPFC and mPFC, which may reflect abnormal cortical inhibition (72). It should be noted, however, that because no evidence directly supporting the relationship between suicide risk and cognitive and emotional processes was collected—especially with regard to death-related cues—these findings should be interpreted with caution.

Suicidal thoughts were also found to be negatively related to connectivity strength between the pCun and pmPFC within the ECN. The pCun plays a crucial role in cognitive and executive functioning as well as memory retrieval (73, 74). Interestingly, a recent study found that individuals with suicidal thoughts exhibited cognitive impairments linked to pronounced pCun activity, including deficits in verbal and working memory and cognitive flexibility (75). Similarly, individuals with a history of suicide attempts showed lower activity in the pCun during a cognitive performance task, reinforcing the connection between pCun dysfunction and suicide risk (76). In addition, individuals with suicidal thoughts demonstrated weak connectivity between the superior frontal gyrus and pCun that was restored following cognitive behavioral therapy (77). A recent study found that brain connectivity between the insula and pCun was reduced in individuals with recent suicidal crises, particularly those with high self-reported impulsivity (13). Collectively, this suggests that reduced brain connectivity involving the pCun is associated with suicide risk and may drive cognitive impairments in these individuals. Although the pmPFC has been less explored in relation to suicide risk, it is known to be involved in error-monitoring (78) and memory integration (79). Our findings suggest that the pCun and pmPFC may serve as potential treatment targets for individuals who have recently experienced a suicidal crisis. Further research is needed to substantiate this hypothesis.

Increased brain connectivity was also seen in the SN, specifically between the lPFC and ParOper and between the mFC and mPC. The lPFC is associated with attention- and goal-seeking behaviors (80), contributing to cognitive control. Reduced connectivity between the lPFC and IFG has been hypothesized to decrease top-down inhibition, thereby escalating suicidal thoughts to attempts and leading to maladaptive decision-making (7). However, studies have reported that increased suicidal thoughts are associated with decreased resting-state activity in the IFG (81) and with reduced connectivity of the dlPFC and the angular gyrus, pCun, and superior frontal gyrus (82). These findings appear somewhat contradictory to our findings regarding the directionality of suicidal thoughts and their neural correlates. Interestingly, youths with suicidal thoughts showed increased brain activation in the lPFC when attempting to regulate emotional responses to negative pictures (60). These findings suggest that the stronger connectivity between the lPFC and ParOper observed in our study may reflect active emotion regulation in individuals with recent suicidal crises, potentially buffering suicidal thoughts from leading to suicidal behaviors.

Connectivity between the mPC and mFC, near the supplementary motor area, has been less explored in the context of suicide. The mPC is associated with navigation and emotional processes (83, 84), while the mFC is involved in decision-making and social processes (85–87), particularly prosocial learning (88). A recent study found that total suicide severity score, as measured by the C-SSRS, was inversely associated with the degree of glucose metabolism in the mPC (89), suggesting that the temporal dynamics of suicidal events might mediate its function. Another recent study found that youths with a high number of suicidal thoughts had increased brain activity in the supplementary motor area and parietal cortex in response to emotionally salient cues (90). Taken together, these studies suggest that the increased connectivity between the mPC and mFC observed in our study may reflect amplified integration of sensory information, which could manifest in decision-making in emotionally challenging situations or social relationships. However, the augmented sensory information integration and decision-making, coupled with a lack of top-down regulation driven by dysfunction in ECN connectivity, may drive maladaptive emotional responses or social behaviors. Further research is required to substantiate these findings and explore their implications.

Broadly, the results of this study on brain connectivity and suicidal thoughts indicate a specific relationship between neural connectivity and SI, with a significant association observed between suicidal thoughts and connectivity between the frontal and parietal brain regions at a macro level. Previous research similarly found that dysfunctional connectivity between these regions plays a crucial role in suicidal behavior and thoughts (91, 92). Specifically, an inverse relationship was observed between connectivity and suicidal thoughts (93, 94) that may have been driven by volumetric alterations in brain regions such as the prefrontal cortex, anterior cingulate, and superior temporal gyrus (5). Furthermore, the microstructural components of white matter, such as the longitudinal fasciculus, and their relationship with suicide risk may help explain the connectivity between the frontal and parietal regions (95, 96). However, our findings also suggest that connectivity from brain regions in close proximity but located in different canonical networks show an opposite directional relationship with suicidal thoughts. This indicates heterogeneous function of brain regions in a network-specific manner, underscoring the importance of understanding network-oriented neural connectivity in the context of SI. A sensitivity analysis (see Supplement) revealed that, with few exceptions, selected DMN connectivity features remained relatively robust to potential confounders, including recent and cumulative suicide burden (measured by the C-SSRS), clinical severity (measured by the MADRS and HAM-A), and temporal measurement error. However, several ECN and SN connectivity features showed attenuation when severity of depressive and anxiety symptoms and medication history were included as covariates. Therefore, while DMN connectivity features may specifically reflect recent SI (measured by the SSI), the ECN and SN connectivity features should be interpreted with caution.

While these preliminary findings are promising and innovative, several limitations warrant consideration. First, the relatively small sample size limits the generalizability and interpretability of our results. Larger longitudinal studies are necessary to confirm these findings. Second, the current study did not include validation tests using external datasets, which are critical for establishing generalizability. Third, participants were either medication-free or maintained on prescribed medications as clinically indicated, which may have introduced variability into the observed associations. Fourth, although most participants in the HR, LR, and CC groups were diagnosed with major depressive disorder, a subset had bipolar disorder, which may have impacted the relationship between suicide risk and neural activity. Fifth, while the DIABLO model successfully differentiated groups with varying degrees of suicide risk, these group differences may have been influenced by confounding variables such as severity of depressive or anxiety symptoms as well as medication history. Future studies should aim to recruit groups with comparable levels of depression and anxiety as well as medication history to more precisely examine the relationship between brain connectivity and suicide risk. Lastly, our findings may have been influenced by the choice of brain parcellation scheme, as different atlases can yield varying connectivity patterns. Future studies should validate these findings across different parcellation schemes to establish their robustness and generalizability.

## 5 Conclusion

This study underscored the potential of the DIABLO method for analyzing electrophysiological MEG data. The results suggest that individuals experiencing a recent suicidal crisis exhibited dysfunction within the DMN, ECN, and SN. Moreover, a subset of brain connectivity scores within these networks was significantly associated with current suicidal thoughts. Our MEG-based within-network connectivity patterns provide objective neurophysiological markers that can distinguish a recent suicidal crisis from historical suicide risk using electrophysiological measures, representing a significant advance towards developing biomarkers that could inform precision psychiatry approaches for suicide prevention. By demonstrating that a recent suicidal crisis has distinct electrophysiological signatures compared to historical risk, our findings support the critical importance of temporal specificity in suicide research and clinical assessment, which can be foundational for longitudinal studies that track individuals across risk states. In addition, our findings revealed that different canonical networks show distinct patterns in a recent suicidal crisis, with the DMN/SN showing positive associations and the ECN showing negative associations with SI. This suggests that the brain connectivity approach yields network-specific therapeutic targets. Lastly, our methodological approach using DIABLO’s multi-block integration framework to combine neurophysiological brain connectivity measures provides a novel analytic approach for future studies that seek to integrate multiple neurobiological measures across different domains, which would advance computational psychiatry research.

## Data Availability

The raw data supporting the conclusions of this article will be made available by the authors, without undue reservation.
