# Supplementary material for: Brain network-driven predictive neural markers for suicide risk
Source: Front Psychiatry. 2026 Oct 20;17:1950068. doi: 10.3389/fpsyt.2026.1950068 (PMC13727727; doi:10.3389/fpsyt.2026.1950068)
Supplement: Supplementary file 1 [file SupplementaryFile1.docx]

*Lee et al – Brain network-driven predictive neural markers for suicide risk using DIABLO*

**Supplement**

**Supplementary Methods**

*Neuroimaging acquisition: Magnetoencephalography (MEG)*

One or two eight-minute eyes-closed resting-state MEG scans were collected during the scanning session. Neuromagnetic data were collected using a CTF 275-channel whole-head system using first-order axial gradiometer MEG sensors and superconducting quantum interference devices (VSM MedTech Ltd., Coquitlam, BC, Canada). Data were collected at 1200 Hz with a 0-300 Hz bandwidth, and synthetic third-order balancing was used to attenuate background environmental noise. In addition, T1-weighted magnetic resonance imaging scans were collected from each participant in a separate scanning session and co-registered to the MEG images using fiducial markers placed at the nasion, left, and right preauricular locations. MEG data preprocessing was performed using the computational resources of the NIH Biowulf high-performance computing cluster (http://hpc.nih.gov).

*Resting-state image processing*

Resting-state MEG data were processed using MNE python (ver 1.3) [1]. The forward and inverse solutions for the data were extracted in the source space using MNE python [2], and the transformation matrix was extracted using the calc_mnettans.py function in the nih_to_mne module. The realistic head boundary element method model (BEM) solution was generated using the FreeSurfer watershed algorithm [3; 4]. The estimated inverse solution was segmented into five-second epochs and projected onto each voxel of the brain within multiple bandwidths ranging from 2 to 58 Hz. The projected MEG power was then segmented into 400 Schaefer atlas parcels across 17 networks [5]. These segmented MEG power parcels were orthogonalized, and pairwise envelope correlation coefficients of those parcels were estimated with the Hilbert transform. The correlation coefficients between parcels located within the same networks, including the default mode network (DMN), executive control network (ECN), and salience network (SN), were defined as intra-network brain connectivity and were used for follow-up analyses.

Quality control included principal component analysis (PCA) using the epPCA method [6] to detect sample outliers. Euclidean distances of all samples from the cluster centroid were calculated, and the three-standard deviation exclusion criterion was applied across canonical networks as follows: (√[(PCA1-PCA1cent)^2^+(PCA2-PCA2cent)^2^]). One sample was identified as an extreme outlier (DMN: distance = 62.95/ M_dist_ = 18.80/ SD_dist_ = 12.57/ 3SD threshold = 56.49; ECN: distance = 59.75/ M_dist_ = 11.82/ SD_dist_ = 9.93/ 3SD threshold = 41.61; SN: distance = 50.87/ M_dist_ =12.19/ SD_dist_ =10.04/ 3SD threshold = 42.31) and was removed prior to downstream analyses. After exclusion, 77 samples remained for subsequent statistical modeling.

*DIABLO analysis*

DIABLO in the mixOmics package in R [7] was used to identify neural connectivity that might differentiate between participant groups. The DIABLO framework integrates multiple high-dimensional datasets in a supervised analysis to pinpoint a limited number of correlated variables that can predict specific outcomes, which is particularly valuable when dealing with more variables than samples. When variables exceed sample size, traditional models often fail to converge and produce unstable predictions. DIABLO addresses this challenge by implementing sparse Partial Least Squares Discriminant Analysis (sPLS-DA), which is specifically designed for high-dimensional data with small sample sizes and uses automatic feature selection and regularization, making it resilient to dimensionality issues [7]. DIABLO extends sparse generalized canonical correlation analysis into a supervised learning framework, building upon sparse partial least squares (sPLS). Initially, the overall correlational structure between different brain networks was estimated using the sPLS approach. The estimated correlation from the first component of each sPLS model guided data integration in DIABLO. The design matrix, a Q × Q matrix with values ranging from 0 to 1, represented the degree of correlation each dataset should have in the DIABLO algorithm. The optimal number of components, which represents the dimensions that maximally explain group membership (similar to principal components in the Principal Components Analysis), was determined by using perf function. The optimal number of within connectivity features from each canonical network (DMN, ECN, and SN) that contribute to group differences was selected through an iterative grid search with leave-one-out cross-validations (LOOCV) using tune.block.splsda function, which was chosen due to the small sample size in the high-risk (HR) group (n=8).

The final analytical component comprised a model that identified a limited number of features across data types exhibiting high correlation with each other, providing insights into which different types of data are relevant and how features from different types interact. To validate our results, permutation testing (999 iterations, RVAideMemoire package in R [8]) was conducted to assess the statistical significance of classification performance against chance-level performance [9], and group differences were further assessed using perf (10 repeated LOOCV) and auroc functions for Receiver Operating Characters (ROC) curves. Benjamini-Hochberg False Discovery Rate (FDR) correction was used for multiple comparisons. For the series of ROC curve scores, the multiple comparison correction was applied with each model, and canonical network was treated as a family of comparisons. Although the small HR group size is a limitation, DIABLO's sparse framework, combined with permutation validation, provides more reliable estimates than traditional machine learning approaches under these constraints.

Resting-state brain connectivity metrics from three brain networks were incorporated, resulting in a final dataset that included 2,628 features of the DMN, 1,225 features of the ECN, and 1,176 features of the SN. The DIABLO framework (mixOmics package in R, version 6.30.0) [10] was then applied to integrate these resting-state brain connectivity measures. DIABLO extends canonical correlation analysis (CCA) and Projection to Latent Structures (PLS) by constructing principal components across blocks to maximize covariance without considering the outcome variable Y. This process results in a signature of correlated variables across multiple datasets measured in the same individuals and discriminates the outcome of interest. The procedure requires tuning three parameters: the design matrix, the number of components, and the number of variables in each dataset to include in the final model. The resulting circos plot is built on a similarity matrix (an approximation of a Pearson correlation matrix), where each entry is calculated as the correlation between the projections of two variables on the principal component. The design matrix specifies how datasets should be correlated, with values between zero (not correlated) and one (fully correlated). Here, the design matrix was tuned by building multi-block PLS models on resting-state brain connectivity data.

*DIABLO: sPLS approach*

The first DIABLO model analysis was conducted using the sPLS approach, which accounts for differences among networks while treating the similarity between networks as weights for explaining the groups. Strong correlations were observed between the networks, and these correlation scores were entered as "weights" in the design matrix. However, this model could not successfully differentiate between the networks, even after 999 permutations (*p*=.319). The performance check conducted using area under the curve (AUC) with 10 repeated LOOCV indicated that the model did not effectively distinguish between the HR and minimal risk (MR) groups and the other groups (Supplementary Table S2).

*DIABLO: Discrimination approach*

A DIABLO model analysis was subsequently conducted that used a discriminant approach, setting the nonorthogonal value in the design matrix to 0.1. This approach successfully differentiated between the networks with 999 permutations (*p*=.018). The discriminative contributions of each neural correlation across networks were specified. Model performance, evaluated by AUC with 10 repeated LOOCV, showed group differentiation (Supplementary Table S2). MANOVA tests were conducted to validate whether the given neural connectivity scores predicted group differences; sex and age were entered as covariates. The groups differed in terms of brain connectivity within the DMN (*F*(3,71)=1.67, *p*=.006, partial η^2^=.40), ECN (*F*(3,71)=1.75, *p*=.001, partial η^2^=.58), and SN (*F*(3,71)=1.64, *p*<.001, partial η^2^=.55). This approach then identified a set of influential features that could predict group differences. To further refine these features, lasso regression models for the DIABLO-selected neural connectivity of two components were conducted in each network. Subsets of neural correlates identified significant differences among the groups: 14 out of 19 for the DMN (component=1), 11 out of 17 for the ECN (component=1), 5 out of 6 for the SN (component=1), 1 out of 2 for the DMN (component=2), 1 out of 15 for the ECN (component=2), and 8 out of 21 for the SN (component=2) (*ps*<.05). A series of ANCOVA analyses was then performed to examine whether selected neural correlates differed between the groups, entering sex and age as covariates.

**Supplementary Results**

*Descriptive statistics: Group Differences*

The lower risk (LR), clinical control (CC), and MR groups all had significantly lower Montgomery-Asberg Depression Rating Scale (MADRS) scores than the HR group (LR: mean difference (MD)=-14.44, *p*<.001; CC: MD=-9.48, *p*=.041; MR: MD=-30.42, *p*<.001). For the Hamilton Depression Rating Scale (HAM-D24), the LR (MD=-10.40, *p*=.003) and MR (MD=-24.08, *p*<.001) groups had lower scores than the HR group, but there was no significant difference compared to the CC group (MD=-5.98, *p*=.181). With regard to the HAM-A, the MR group had significantly lower scores than the HR group (MD=-15.83, *p*<.001), but no significant difference was observed between the HR and LR groups (MD=-4.12, *p*=.271) or between the HR and CC groups (MD=-2.59, *p*=.676). Primary diagnoses varied significantly across groups. Specifically, the CC group had significantly higher rates of major depressive disorder as the primary diagnosis than either the HR (p=.001) or LR (p=.010) groups, while no such difference was seen between the HR and LR groups (p=.410). The temporal interval between MEG and clinical assessments, including the MADRS, HAM-D24, HAM-A, and Scale for Suicide Ideation (SSI) varied across groups, and the HR group had significantly more delays between measures than the LR (MD=2.78, *p*=.027), CC (MD=2.76, *p*=.023), and MR (MD=3.18, *p*=.009) groups.

*DIABLO analysis: Group Differences*

Only meaningful differences in neural correlates between the HR and other groups, with alpha=0.05, are reported here (Supplementary Tables S3 and S4). In the DMN, the HR group showed reduced brain connectivity between the left dorsolateral prefrontal cortex (dlPFC) and the left retrosplenial cortex and right medial prefrontal cortex (mPFC) as well as between the right dlPFC and the left joint area of the precuneus and posterior cingulate cortex (pCun/PCC) compared to the LR group (*ps*<.05). In addition, compared to the MR group, the HR group showed reduced brain connectivity between the right dlPFC and the left pCun/PCC (*ps*<.05). These findings suggest downregulated communication between the frontal and medial-parietal brain regions within the DMN for the HR group.

In the ECN, the HR group had reduced brain connectivity between the left medial/posterior prefrontal cortex and the left ventrolateral prefrontal cortex (vlPFC) and left precuneus (pCun) and between different segments of the dlPFC compared to the LR group (*ps*<.05). The HR group also had reduced brain connectivity between the vlPFC and the right parietal cingulate cortex compared to the CC group (*ps*<.05). These findings suggest downregulated communication within the frontal regions and between the frontal and medial-parietal brain regions, such as the pCun, in the HR group.

In the SN, the HR group had reduced brain connectivity between the right medial frontal cortex (mFC) and the left insula and right lateral prefrontal cortex (lPFC) compared to the LR group (*ps*<.05). The HR group also had reduced brain connectivity between the left mFC and the medial parietal cortex (mPC) and between the right lPFC and the right inferior parietal lobule compared to the CC group (*ps*<.05). In addition, the HR group showed enhanced brain connectivity between the left lPFC and the left parietal operculum (ParOper) compared to the CC group (*ps*<.05). Lastly, the HR group had reduced brain connectivity between the left mFC and the left and right lPFC, right mPC, and left insula compared to the MR group (*ps*<.05). These findings suggest downregulated communication within the frontal regions and between the frontal and sensory- and emotion-related regions, insula, and inferior parietal lobule.

*DIABLO: Sensitivity Analyses*

To conduct sensitivity analyses for models showing significant differences in the HR group compared to the other groups, potential confounding variables were included as covariates, including medication history (0=no, 1=yes); poly-medication history (0=no drug, 1=single drug, 2=poly drugs); the severity of depressive and anxiety symptoms, as measured by the MADRS and Hamilton Anxiety Rating Scale (HAM-A), respectively; history of recent suicide attempts (0=no, 1=yes); and the temporal interval between MEG and clinical data collection. Columbia Suicide Severity Rating Scale (C-SSRS)-derived suicide measures, including recent suicidal ideation (<2weeks), history of past suicide attempts, and total suicide attempt frequency were also examined. With few exceptions, most analyses indicated significant interactions between selected connectivity strengths and recent suicidal ideation scores measured by the SSI. ECN connectivity between the posterior mFC and precuneus was suppressed by medication history (95% CrI: -1.11, 0.01), poly-medication history (95% CrI: -1.05, 0.02), and severity of anxiety symptoms measured by the HAM-A (95% CrI: -0.01, 0.01) in the HR versus MR groups, and by poly-medication history (95% CrI: -1.02, 0.04), history of recent suicide attempts (95% CrI: -1.05, 0.06), and temporal interval between MEG and clinical assessments (95% CrI: -1.05, 0.05) in the HR versus MR groups. SN connectivity between the parietal operculum and lateral prefrontal cortex was suppressed by medication history (95% CrI: -0.01, 0.78), severity of depressive symptoms as measured by the MADRS (95% CrI: 0.00, 0.72), and history of recent suicide attempts (95% CrI: 0.00, 0.69) in the HR versus CC groups. SN connectivity between the mFC and mPC was also suppressed by history of past suicide attempts in the SR group compared to the MR (95% CrI: -0.01, 1.14) and LR (95% CrI: -0.01, 1.10) groups. These findings suggest that the observed connectivity differences could partially be explained by medication effects, clinical severity, and acute and cumulative suicide burden rather than recent SI alone. Within-network connectivities showed robust associations with recent SI across DMN, ECN, and SN networks, maintaining consistent direction and magnitude regardless of covariate inclusion, even when statistical significance was not achieved for the association between within-network connectivity and recent SI.

**Supplementary References**

1. Gramfort A, Luessi M, Larson E, Engemann DA, Strohmeier D, Brodbeck C, et al. MEG and EEG data analysis with MNE-Python. *Front Neurosci* (2013) 7: 267.

2. Gramfort A, Luessi M, Larson E, Engemann DA, Strohmeier D, Brodbeck C, et al. MNE software for processing MEG and EEG data. *Neuroimage* (2014) 86: 446-460.

3. Dale AM, and Sereno MI. Improved localization of cortical activity by combining EEG and MEG with MRI cortical surface reconstruction: a linear approach. *J Cogn Neurosci* (1993) 5: 162-176.

4. Fischl B, Salat DH, van der Kouwe AJ, Makris N, Ségonne F, Quinn BT, et al. Sequence-independent segmentation of magnetic resonance images. *Neuroimage* (2024) 23 (Suppl 1): S69-S84.

5. Schaefer A, Kong R, Gordon EM, Laumann TO, Zuo X-N, Holmes AJ, et al. Local-global parcellation of the human cerebral cortex from intrinsic functional connectivity MRI. *Cereb Cortex* (2018) 28: 3095-3114.

6. Beaton D, Fatt CRC, and Abdi H. An ExPosition of multivariate analysis with the singular value decomposition in R. *Comput Stat Data Anal* (2014) 72: 176-189.

7. Rohart F, Gautier B, Singh A, and Lê Cao K-A. mixOmics: An R package for ‘omics feature selection and multiple data integration. *PLoS Comput Biol* (2017) 13: e1005752.

8. Herve M, RVAideMemoire: Testing and Plotting Procedures for Biostatistics. R package version 0.9-83-11. Available at: <https://CRAN.R-project.org/package=RVAideMemoire>, 2025.

9. Westerhuis JA, Hoefsloot HCJ, Smit S, Vis DJ, Smilde AK, van Velzen EJJ, et al. Assessment of PLSDA cross validation. *Metabolomics* (2008) 4: 81-89.

10. Singh A, Shannon CP, Gautier B, Rohart F, Vacher M, Tebbutt SJ, et al. DIABLO: an integrative approach for identifying key molecular drivers from multi-omics assays. *Bioinformatics* (2019) 35: 3055-3062.

**Supplementary Table S1. Correlation between the clinical scales**

|  | **1** | **2** | **3** | **4** | **5** |
| --- | --- | --- | --- | --- | --- |
| Age |  |  |  |  |  |
| MADRS | 0.08 (.334) |  |  |  |  |
| HAM-D24 | 0.08 (.357) | 0.83 (<.001) |  |  |  |
| HAM-A | 0.11 (.228) | 0.73 (<.001) | 0.79 (<.001) |  |  |
| Current Suicidal Ideation** | 0.13 (.172) | 0.46 (<.001) | 0.49 (<.001) | 0.42 (<.001) |  |
| Past Suicidal Ideation** | 0.03 (.694) | 0.36 (<.001) | 0.38 (<.001) | 0.39 (<.001) | 0.37 (<.001) |

**Both past and current suicide ideation were measured using the Scale for Suicide Ideation (SSI).

HAM-A: Hamilton Anxiety Rating Scale; MADRS: Montgomery-Åsberg Depression Rating Scale; HAM-D24: Hamilton Rating Scale for Depression-24. Scores are based on Kendall’s Rho values. *P*-values are reported in parentheses.

**Supplementary Table S2. Receiver operating characteristics (ROC) curve scores in the DIABLO model in the canonical networks for the first and second components.**

| **Model** | **Component** | **Network** | **Group Comparison** | **AUC** | ***p*-value** | **FDR**  ***p*-value** |
| --- | --- | --- | --- | --- | --- | --- |
| sPLS | 1 | DMN | HR group vs. Others | 0.77 | .014 | .019 |
|  | 1 | DMN | LR group vs. Others | 0.78 | <.001 | .002 |
|  | 1 | DMN | CC group vs. Others | 0.74 | .001 | .002 |
|  | 1 | DMN | MR group vs. Others | 0.56 | .444 | .507 |
|  | 2 | DMN | HR group vs. Others | 0.78 | .009 | .014 |
|  | 2 | DMN | LR group vs. Others | 0.83 | <.001 | .002 |
|  | 2 | DMN | CC group vs. Others | 0.85 | <.001 | .002 |
|  | 2 | DMN | MR group vs. Others | 0.54 | .567 | .567 |
|  | 1 | ECN | HR group vs. Others | 0.65 | .171 | .228 |
|  | 1 | ECN | LR group vs. Others | 0.79 | <.001 | .002 |
|  | 1 | ECN | CC group vs. Others | 0.81 | <.001 | .002 |
|  | 1 | ECN | MR group vs. Others | 0.56 | .450 | .514 |
|  | 2 | ECN | HR group vs. Others | 0.76 | .017 | .027 |
|  | 2 | ECN | LR group vs. Others | 0.79 | <.001 | .002 |
|  | 2 | ECN | CC group vs. Others | 0.82 | <.001 | .002 |
|  | 2 | ECN | MR group vs. Others | 0.54 | .575 | .575 |
|  | 1 | SN | HR group vs. Others | 0.76 | .017 | .025 |
|  | 1 | SN | LR group vs. Others | 0.73 | .001 | .003 |
|  | 1 | SN | CC group vs. Others | 0.73 | .002 | .004 |
|  | 1 | SN | MR group vs. Others | 0.60 | .185 | .185 |
|  | 2 | SN | HR group vs. Others | 0.75 | .019 | .025 |
|  | 2 | SN | LR group vs. Others | 0.80 | <.001 | .003 |
|  | 2 | SN | CC group vs. Others | 0.81 | <.001 | .003 |
|  | 2 | SN | MR group vs. Others | 0.60 | .181 | .185 |
|  | 1 | Overall | HR group vs. Others | 0.70 | .135 | .180 |
|  | 1 | Overall | LR group vs. Others | 0.74 | .009 | .018 |
|  | 1 | Overall | CC group vs. Others | 0.75 | .006 | .016 |
|  | 1 | Overall | MR group vs. Others | 0.58 | .366 | .366 |
|  | 2 | Overall | HR group vs. Others | 0.75 | .045 | .072 |
|  | 2 | Overall | LR group vs. Others | 0.79 | .005 | .016 |
|  | 2 | Overall | CC group vs. Others | 0.80 | .003 | .016 |
|  | 2 | Overall | MR group vs. Others | 0.61 | .226 | .258 |
| Discriminant | 1 | DMN | HR group vs. Others | 0.78 | .010 | .016 |
|  | 1 | DMN | LR group vs. Others | 0.81 | <.001 | .002 |
|  | 1 | DMN | CC group vs. Others | 0.84 | <.001 | .002 |
|  | 1 | DMN | MR group vs. Others | 0.64 | .061 | .062 |
|  | 2 | DMN | HR group vs. Others | 0.77 | .012 | .016 |
|  | 2 | DMN | LR group vs. Others | 0.87 | <.001 | .002 |
|  | 2 | DMN | CC group vs. Others | 0.91 | <.001 | .002 |
|  | 2 | DMN | MR group vs. Others | 0.64 | .062 | .062 |
|  | 1 | ECN | HR group vs. Others | 0.72 | .042 | .055 |
|  | 1 | ECN | LR group vs. Others | 0.82 | <.001 | .002 |
|  | 1 | ECN | CC group vs. Others | 0.89 | <.001 | .002 |
|  | 1 | ECN | MR group vs. Others | 0.65 | .048 | .055 |
|  | 2 | ECN | HR group vs. Others | 0.82 | .003 | .005 |
|  | 2 | ECN | LR group vs. Others | 0.90 | <.001 | .002 |
|  | 2 | ECN | CC group vs. Others | 0.98 | <.001 | .002 |
|  | 2 | ECN | MR group vs. Others | 0.64 | .055 | .055 |
|  | 1 | SN | HR group vs. Others | 0.79 | .008 | .011 |
|  | 1 | SN | LR group vs. Others | 0.74 | .001 | .002 |
|  | 1 | SN | CC group vs. Others | 0.81 | <.001 | .002 |
|  | 1 | SN | MR group vs. Others | 0.68 | .013 | .015 |
|  | 2 | SN | HR group vs. Others | 0.80 | .006 | .010 |
|  | 2 | SN | LR group vs. Others | 0.86 | <.001 | .002 |
|  | 2 | SN | CC group vs. Others | 0.94 | <.001 | .002 |
|  | 2 | SN | MR group vs. Others | 0.68 | .018 | .018 |
|  | 1 | Overall | HR group vs. Others | 0.76 | .024 | .032 |
|  | 1 | Overall | LR group vs. Others | 0.79 | <.001 | .002 |
|  | 1 | Overall | CC group vs. Others | 0.85 | <.001 | .002 |
|  | 1 | Overall | MR group vs. Others | 0.66 | .047 | .047 |
|  | 2 | Overall | HR group vs. Others | 0.81 | .007 | .011 |
|  | 2 | Overall | LR group vs. Others | 0.87 | <.001 | .002 |
|  | 2 | Overall | CC group vs. Others | 0.95 | <.001 | .002 |
|  | 2 | Overall | MR group vs. Others | 0.66 | .046 | .047 |

DIABLO: Data Integration Analysis for Biomarker discovery using Latent variable approaches for Omics studies; AUC: area under the curve; DMN: default mode network; ECN: executive control network; SN: salience network; HR: high risk group; LR: lower risk group; CC: clinical controls; MR: minimal risk group; sPLS: sparse partial least squares; FDR: false discovery rate.

**Supplementary Table S3. MANOVA results of brain connectivity scores in the canonical networks for the first and second components**

| **Network** | **Comp-onent** | **Label** | **F-score** | ***p*-value** | **FDR**  ***p*-value** |
| --- | --- | --- | --- | --- | --- |
| DMN | 1 | LH_DefaultA_pCunPCC_3.lh.LH_DefaultA_pCunPCC_7.lh | 4.91 | 0.004 | .006 |
| DMN | 1 | LH_DefaultA_PFCm_4.lh.LH_DefaultB_PFCd_6.lh | 6.15 | <.001 | .003 |
| DMN | 1 | LH_DefaultB_PFCd_6.lh.LH_DefaultB_PFCv_5.lh | 6.56 | <.001 | .002 |
| DMN | 1 | LH_DefaultB_IPL_2.lh.LH_DefaultC_PHC_1.lh | 5.58 | 0.002 | .004 |
| DMN | 1 | LH_DefaultA_PFCd_1.lh.LH_DefaultC_Rsp_3.lh | 6.48 | <.001 | .002 |
| DMN | 1 | LH_DefaultB_PFCd_2.lh.LH_DefaultC_Rsp_3.lh | 6.76 | <.001 | .002 |
| DMN | 1 | RH_DefaultA_PFCd_2.rh.RH_DefaultA_PFCm_3.rh | 5.10 | 0.003 | .005 |
| DMN | 1 | LH_DefaultB_PFCd_5.lh.RH_DefaultA_PFCm_4.rh | 6.64 | <.001 | .002 |
| DMN | 1 | LH_DefaultB_PFCv_2.lh.RH_DefaultA_PFCm_6.rh | 4.77 | 0.004 | .006 |
| DMN | 1 | LH_DefaultA_pCunPCC_2.lh.RH_DefaultB_PFCd_3.rh | 8.53 | <.001 | .001 |
| DMN | 1 | LH_DefaultA_pCunPCC_2.lh.RH_DefaultB_PFCd_4.rh | 5.60 | 0.002 | .004 |
| DMN | 1 | LH_DefaultA_pCunPCC_7.lh.RH_DefaultB_PFCd_4.rh | 6.56 | <.001 | .002 |
| DMN | 1 | LH_DefaultB_PFCd_6.lh.RH_DefaultB_PFCd_4.rh | 4.32 | 0.007 | .008 |
| DMN | 1 | LH_DefaultA_PFCm_3.lh.RH_DefaultB_PFCv_1.rh | 5.02 | 0.003 | .005 |
| ECN | 1 | LH_ContB_PFClv_3.lh.LH_ContB_PFCmp_1.lh | 7.23 | <.001 | .002 |
| ECN | 1 | LH_ContB_PFCmp_1.lh.LH_ContC_pCun_1.lh | 8.31 | <.001 | .002 |
| ECN | 1 | LH_ContA_PFCd_1.lh.RH_ContA_IPS_1.rh | 5.29 | 0.002 | .011 |
| ECN | 1 | LH_ContB_PFClv_2.lh.RH_ContA_IPS_1.rh | 7.14 | <.001 | .002 |
| ECN | 1 | LH_ContA_PFClv_1.lh.RH_ContB_IPL_4.rh | 5.51 | 0.002 | .010 |
| ECN | 1 | LH_ContC_pCun_1.lh.RH_ContB_PFCld_2.rh | 7.39 | <.001 | .002 |
| ECN | 1 | RH_ContB_PFCld_1.rh.RH_ContB_PFCld_2.rh | 4.60 | 0.005 | .016 |
| ECN | 1 | LH_ContB_IPL_3.lh.RH_ContB_PFCld_4.rh | 6.41 | <.001 | .004 |
| ECN | 1 | LH_ContA_PFCl_3.lh.RH_ContB_PFClv_2.rh | 4.47 | 0.006 | .017 |
| ECN | 1 | LH_ContB_PFCmp_1.lh.RH_ContB_PFClv_2.rh | 5.05 | 0.003 | .013 |
| ECN | 1 | LH_ContC_Cingp_2.lh.RH_ContB_PFCmp_1.rh | 3.63 | 0.017 | .034 |
| SN | 1 | LH_SalVentAttnA_FrMed_2.lh.LH_SalVentAttnB_PFCl_2.lh | 5.86 | 0.001 | .011 |
| SN | 1 | LH_SalVentAttnA_Ins_3.lh.RH_SalVentAttnA_FrMed_1.rh | 4.63 | 0.005 | .029 |
| SN | 1 | RH_SalVentAttnA_FrMed_1.rh.RH_SalVentAttnB_PFCl_2.rh | 4.60 | 0.005 | .029 |
| SN | 1 | RH_SalVentAttnA_FrMed_1.rh.RH_SalVentAttnB_PFCl_3.rh | 9.63 | <.001 | .001 |
| SN | 1 | RH_SalVentAttnA_Ins_3.rh.RH_SalVentAttnB_PFCl_3.rh | 4.20 | 0.009 | .033 |
| DMN | 2 | LH_DefaultB_PFCv_5.lh.LH_DefaultC_PHC_3.lh | 4.32 | 0.007 | .009 |
| ECN | 2 | LH_ContB_PFClv_3.lh.RH_ContC_Cingp_2.rh | 4.05 | 0.010 | .023 |
| SN | 2 | LH_SalVentAttnA_Ins_1.lh.LH_SalVentAttnA_ParMed_2.lh | 2.56 | 0.060 | .129 |
| SN | 2 | LH_SalVentAttnA_ParOper_2.lh.LH_SalVentAttnB_PFCl_3.lh | 7.59 | <.001 | .002 |
| SN | 2 | LH_SalVentAttnA_ParMed_2.lh.RH_SalVentAttnA_FrOper_2.rh | 2.31 | 0.084 | .162 |
| SN | 2 | RH_SalVentAttnA_FrMed_3.rh.RH_SalVentAttnA_Ins_4.rh | 1.66 | 0.184 | .238 |
| SN | 2 | LH_SalVentAttnA_FrMed_3.lh.RH_SalVentAttnA_ParMed_2.rh | 3.31 | 0.025 | .068 |
| SN | 2 | RH_SalVentAttnA_Ins_2.rh.RH_SalVentAttnA_PrC_1.rh | 2.62 | 0.057 | .129 |
| SN | 2 | LH_SalVentAttnA_ParMed_2.lh.RH_SalVentAttnB_IPL_1.rh | 3.61 | 0.017 | .059 |
| SN | 2 | RH_SalVentAttnB_IPL_1.rh.RH_SalVentAttnB_PFCl_2.rh | 3.38 | 0.023 | .068 |

A Ins: anterior insula; Cingp: posterior cingulate cortex; ContA: executive control network type A; ContB: executive control network type B; ContC: executive control network type C; DefaultA: default mode network type A; Default B: default mode network type B; Default C: default mode network type C; DMN: default mode network; ECN: executive control network; FDR: false discovery rate; FrMed: medial frontal cortex; IPL: inferior parietal lobule; IPS: intraparietal sulcus; SalVentAttnA: salience network type A; SalVentAttnB: salience network type B; LH: left hemisphere; ParMed: medial parietal cortex; ParOper: parietal operculum; PCun: precuneus; pCunPCC: precuneus posterior cingulate cortex; PFCd: dorsal prefrontal cortex; PFCl: lateral prefrontal cortex; PFCld: dorsolateral prefrontal cortex; PFClv: ventrolateral prefrontal cortex; PFCm: medial prefrontal cortex; PFCmp: posterior medial prefrontal cortex; PFCv: ventral prefrontal cortex; PHC: parahippocampal cortex; RH: right hemisphere; PrC: precentral cortex; Rsp: retrosplenial cortex; SN: salience network.

**Supplementary Table S4. Mean differences for each brain connectivity score in the canonical networks for the first and second components**

| **Network** | **Comp-onent** | **Tar-get** | **Refer-ence** | **Connectivity** | **Mean Difference** | ***p*- value** | **FDR**  ***p*-value** |
| --- | --- | --- | --- | --- | --- | --- | --- |
| DMN | 1 | HR | LR | LH_DefaultA_PFCd_1.lh.LH_DefaultC_Rsp_3.lh | -0.77 | .003 | .031 |
| DMN | 1 | HR | LR | LH_DefaultB_PFCd_5.lh.RH_DefaultA_PFCm_4.rh | -0.53 | .048 | .165 |
| DMN | 1 | HR | LR | LH_DefaultA_pCunPCC_2.lh.RH_DefaultB_PFCd_3.rh | -0.72 | .004 | .036 |
| DMN | 1 | HR | LR | LH_DefaultA_pCunPCC_2.lh.RH_DefaultB_PFCd_4.rh | -0.73 | .005 | .036 |
| DMN | 1 | HR | LR | LH_DefaultA_pCunPCC_7.lh.RH_DefaultB_PFCd_4.rh | -0.55 | .027 | .105 |
| DMN | 1 | HR | HV | LH_DefaultA_pCunPCC_7.lh.RH_DefaultB_PFCd_4.rh | -0.60 | .017 | .081 |
| DMN | 1 | LR | CC | LH_DefaultA_pCunPCC_3.lh.LH_DefaultA_pCunPCC_7.lh | 0.52 | .003 | .031 |
| DMN | 1 | LR | CC | LH_DefaultA_PFCm_4.lh.LH_DefaultB_PFCd_6.lh | 0.60 | .001 | .031 |
| DMN | 1 | LR | CC | LH_DefaultB_PFCd_6.lh.LH_DefaultB_PFCv_5.lh | 0.54 | .002 | .031 |
| DMN | 1 | LR | CC | LH_DefaultB_IPL_2.lh.LH_DefaultC_PHC_1.lh | 0.58 | .002 | .031 |
| DMN | 1 | LR | CC | LH_DefaultA_PFCd_1.lh.LH_DefaultC_Rsp_3.lh | 0.53 | .005 | .036 |
| DMN | 1 | LR | CC | LH_DefaultB_PFCd_2.lh.LH_DefaultC_Rsp_3.lh | 0.55 | .002 | .031 |
| DMN | 1 | LR | CC | RH_DefaultA_PFCd_2.rh.RH_DefaultA_PFCm_3.rh | 0.51 | .003 | .031 |
| DMN | 1 | LR | CC | LH_DefaultB_PFCd_5.lh.RH_DefaultA_PFCm_4.rh | 0.63 | <.001 | .015 |
| DMN | 1 | LR | CC | LH_DefaultB_PFCv_2.lh.RH_DefaultA_PFCm_6.rh | 0.60 | .002 | .031 |
| DMN | 1 | LR | CC | LH_DefaultA_pCunPCC_2.lh.RH_DefaultB_PFCd_3.rh | 0.65 | <.001 | .015 |
| DMN | 1 | LR | CC | LH_DefaultA_pCunPCC_2.lh.RH_DefaultB_PFCd_4.rh | 0.50 | .007 | .047 |
| DMN | 1 | LR | CC | LH_DefaultA_pCunPCC_7.lh.RH_DefaultB_PFCd_4.rh | 0.42 | .170 | .081 |
| DMN | 1 | LR | CC | LH_DefaultB_PFCd_6.lh.RH_DefaultB_PFCd_4.rh | 0.56 | .007 | .047 |
| DMN | 1 | LR | CC | LH_DefaultA_PFCm_3.lh.RH_DefaultB_PFCv_1.rh | 0.54 | .002 | .031 |
| DMN | 1 | LR | MR | LH_DefaultA_PFCd_1.lh.LH_DefaultC_Rsp_3.lh | 0.43 | .032 | .119 |
| DMN | 1 | CC | MR | LH_DefaultA_pCunPCC_3.lh.LH_DefaultA_pCunPCC_7.lh | -0.42 | .034 | .123 |
| DMN | 1 | CC | MR | LH_DefaultA_PFCm_4.lh.LH_DefaultB_PFCd_6.lh | -0.48 | .020 | .088 |
| DMN | 1 | CC | MR | LH_DefaultB_PFCd_6.lh.LH_DefaultB_PFCv_5.lh | -0.48 | .011 | .058 |
| DMN | 1 | CC | MR | LH_DefaultB_IPL_2.lh.LH_DefaultC_PHC_1.lh | -0.46 | .035 | .124 |
| DMN | 1 | CC | MR | LH_DefaultB_PFCd_2.lh.LH_DefaultC_Rsp_3.lh | -0.55 | .004 | .035 |
| DMN | 1 | CC | MR | LH_DefaultA_pCunPCC_2.lh.RH_DefaultB_PFCd_3.rh | -0.45 | .024 | .099 |
| DMN | 1 | CC | MR | LH_DefaultA_pCunPCC_7.lh.RH_DefaultB_PFCd_4.rh | -0.47 | .010 | .058 |
| ECN | 1 | HR | LR | LH_ContB_PFClv_3.lh.LH_ContB_PFCmp_1.lh | -0.55 | .049 | .333 |
| ECN | 1 | HR | LR | LH_ContB_PFCmp_1.lh.LH_ContC_pCun_1.lh | -0.63 | .005 | .096 |
| ECN | 1 | HR | LR | RH_ContB_PFCld_1.rh.RH_ContB_PFCld_2.rh | -0.64 | .046 | .333 |
| ECN | 1 | LR | CC | LH_ContB_PFClv_3.lh.LH_ContB_PFCmp_1.lh | 0.60 | .001 | .034 |
| ECN | 1 | LR | CC | LH_ContB_PFCmp_1.lh.LH_ContC_pCun_1.lh | 0.58 | <.001 | .011 |
| ECN | 1 | LR | CC | LH_ContA_PFCd_1.lh.RH_ContA_IPS_1.rh | 0.48 | .002 | .042 |
| ECN | 1 | LR | CC | LH_ContB_PFClv_2.lh.RH_ContA_IPS_1.rh | 0.63 | <.001 | .011 |
| ECN | 1 | LR | CC | LH_ContA_PFClv_1.lh.RH_ContB_IPL_4.rh | 0.59 | .001 | .036 |
| ECN | 1 | LR | CC | LH_ContC_pCun_1.lh.RH_ContB_PFCld_2.rh | 0.49 | .007 | .110 |
| ECN | 1 | LR | CC | RH_ContB_PFCld_1.rh.RH_ContB_PFCld_2.rh | 0.50 | .024 | .241 |
| ECN | 1 | LR | CC | LH_ContB_IPL_3.lh.RH_ContB_PFCld_4.rh | 0.54 | .002 | .042 |
| ECN | 1 | LR | CC | LH_ContA_PFCl_3.lh.RH_ContB_PFClv_2.rh | 0.38 | .044 | .333 |
| ECN | 1 | LR | CC | LH_ContB_PFCmp_1.lh.RH_ContB_PFClv_2.rh | 0.62 | .001 | .036 |
| ECN | 1 | LR | CC | LH_ContC_Cingp_2.lh.RH_ContB_PFCmp_1.rh | 0.43 | .025 | .241 |
| ECN | 1 | CC | MR | LH_ContB_PFClv_3.lh.LH_ContB_PFCmp_1.lh | -0.55 | .004 | .082 |
| ECN | 1 | CC | MR | LH_ContC_pCun_1.lh.RH_ContB_PFCld_2.rh | -0.69 | <.001 | .011 |
| ECN | 1 | CC | MR | LH_ContB_IPL_3.lh.RH_ContB_PFCld_4.rh | -0.50 | .008 | .110 |
| ECN | 1 | CC | MR | LH_ContC_Cingp_2.lh.RH_ContB_PFCmp_1.rh | -0.42 | .044 | .333 |
| SN | 1 | HR | LR | LH_SalVentAttnA_Ins_3.lh.RH_SalVentAttnA_FrMed_1.rh | -0.50 | .050 | .351 |
| SN | 1 | HR | LR | RH_SalVentAttnA_FrMed_1.rh.RH_SalVentAttnB_PFCl_2.rh | -0.54 | .026 | .296 |
| SN | 1 | HR | LR | RH_SalVentAttnA_FrMed_1.rh.RH_SalVentAttnB_PFCl_3.rh | -0.68 | .007 | .200 |
| SN | 1 | HR | MR | LH_SalVentAttnA_FrMed_2.lh.LH_SalVentAttnB_PFCl_2.lh | -0.60 | .031 | .311 |
| SN | 1 | HR | MR | LH_SalVentAttnA_Ins_3.lh.RH_SalVentAttnA_FrMed_1.rh | -0.52 | .045 | .335 |
| SN | 1 | HR | MR | RH_SalVentAttnA_FrMed_1.rh.RH_SalVentAttnB_PFCl_3.rh | -0.61 | .023 | .296 |
| SN | 1 | LR | CC | LH_SalVentAttnA_FrMed_2.lh.LH_SalVentAttnB_PFCl_2.lh | 0.42 | .027 | .296 |
| SN | 1 | LR | CC | RH_SalVentAttnA_FrMed_1.rh.RH_SalVentAttnB_PFCl_2.rh | 0.42 | .011 | .266 |
| SN | 1 | LR | CC | RH_SalVentAttnA_FrMed_1.rh.RH_SalVentAttnB_PFCl_3.rh | 0.66 | <.001 | .020 |
| SN | 1 | LR | CC | RH_SalVentAttnA_Ins_3.rh.RH_SalVentAttnB_PFCl_3.rh | 0.47 | .021 | .296 |
| SN | 1 | CC | MR | LH_SalVentAttnA_FrMed_2.lh.LH_SalVentAttnB_PFCl_2.lh | -0.56 | .003 | .115 |
| SN | 1 | CC | MR | RH_SalVentAttnA_FrMed_1.rh.RH_SalVentAttnB_PFCl_3.rh | -0.59 | .001 | .080 |
| DMN | 2 | LR | CC | LH_DefaultB_PFCv_5.lh.LH_DefaultC_PHC_3.lh | -0.50 | .005 | .036 |
| ECN | 2 | HR | CC | LH_ContB_PFClv_3.lh.RH_ContC_Cingp_2.rh | -0.54 | .041 | .333 |
| ECN | 2 | LR | CC | LH_ContB_PFClv_3.lh.RH_ContC_Cingp_2.rh | -0.43 | .017 | .199 |
| SN | 2 | HR | CC | LH_SalVentAttnA_ParOper_2.lh.LH_SalVentAttnB_PFCl_3.lh | 0.71 | .004 | .115 |
| SN | 2 | HR | CC | LH_SalVentAttnA_FrMed_3.lh.RH_SalVentAttnA_ParMed_2.rh | -0.64 | .022 | .296 |
| SN | 2 | HR | CC | RH_SalVentAttnB_IPL_1.rh.RH_SalVentAttnB_PFCl_2.rh | -0.60 | .037 | .315 |
| SN | 2 | HR | MR | LH_SalVentAttnA_FrMed_3.lh.RH_SalVentAttnA_ParMed_2.rh | -0.65 | .020 | .296 |
| SN | 2 | LR | CC | LH_SalVentAttnA_ParOper_2.lh.LH_SalVentAttnB_PFCl_3.lh | 0.61 | <.001 | .020 |
| SN | 2 | LR | MR | LH_SalVentAttnA_ParMed_2.lh.RH_SalVentAttnB_IPL_1.rh | -0.40 | .035 | .311 |
| SN | 2 | CC | MR | LH_SalVentAttnA_ParOper_2.lh.LH_SalVentAttnB_PFCl_3.lh | -0.40 | .042 | .335 |

A Ins: anterior insula; Cingp: posterior cingulate cortex; CC: clinical controls; ContA: executive control network type A; ContB: executive control network type B; ContC: executive control network type C; DefaultA: default mode network type A; Default B: default mode network type B; Default C: default mode network type C; DMN: default mode network; ECN: executive control network; FDR: false discovery rate; FrMed: medial frontal cortex; HR: high risk group; IPL: inferior parietal lobule; IPS: intraparietal sulcus; LH: left hemisphere; LR: lower risk group; MR: minimal-risk group; ParMed: medial parietal cortex; ParOper: parietal operculum; PCun: precuneus; pCunPCC: precuneus posterior cingulate cortex; PFCd: dorsal prefrontal cortex; PFCl: lateral prefrontal cortex; PFCld: dorsolateral prefrontal cortex; PFClv: ventrolateral prefrontal cortex; PFCm: medial prefrontal cortex; PFCmp: posterior medial prefrontal cortex; PFCv: ventral prefrontal cortex; PHC: parahippocampal cortex; RH: right hemisphere; Rsp: retrosplenial cortex; SalVentAttnA: salience network type A; SalVentAttnB: salience network type B; SN: salience network.

**Supplementary Table S5. MNI coordinates of brain regions associated with recent suicidal ideation**

| **Network** | **Label (as per [5])** | **Hemisphere** | **Connectivity** | **MNI Coordinates** | | |
| --- | --- | --- | --- | --- | --- | --- |
|  |  |  |  | **X** | **Y** | **Z** |
| DMN | LH_DefaultA_pCunPCC_2 | Left | Posterior precuneus/PCC (posterior pCun/PCC) | -5 | -60 | 30 |
| DMN | LH_DefaultA_pCunPCC_7 | Left | Precuneus/PCC (pCun/PCC) | -7 | -51 | 43 |
| DMN | RH_DefaultA_PFCm_4 | Right | Medial prefrontal cortex (mPFC) | 7 | 54 | 13 |
| DMN | LH_DefaultB_PFCd_5 | Left | Dorsolateral prefrontal cortex (dlPFC) | -13 | 24 | 61 |
| DMN | RH_DefaultB_PFCd_4 | Right | Dorsolateral prefrontal cortex (dlPFC) | 14 | 39 | 52 |
| ECN | LH_ContB_PFCmp_1 | Left | Posterior medial prefrontal cortex (pmPFC) | -4 | 28 | 47 |
| ECN | LH_ContC_pCun_1 | Left | Precuneus (pCun) | -10 | -70 | 32 |
| SN | LH_SalVentAttnA_FrMed_3 | Left | Medial frontal cortex (mFC) | -8 | -3 | 71 |
| SN | RH_SalVentAttnA_ParMed_2 | Right | Medial parietal cortex (mPC) | 12 | -34 | 43 |
| SN | LH_SalVentAttnA_ParOper_2 | Left | Parietal operculum (ParOper) | -58 | -44 | 27 |
| SN | LH_SalVentAttnB_PFCl_3 | Left | Lateral prefrontal cortex (lPFC) | -36 | 32 | 38 |

ContA: executive control network type A; ContB: executive control network type B; ContC: executive control network type C; DefaultA: default mode network type A; Default B: default mode network type B; Default C: default mode network type C; DMN: default mode network; ECN: executive control network; FrMed: medial frontal cortex; LH: left hemisphere; MNI: Montreal Neurological Institute; ParMed: medial parietal cortex; ParOper: parietal operculum; pCun: precuneus; PCunPCC: precuneus posterior cingulate cortex; PFCl: lateral prefrontal cortex; PFCld: dorsolateral prefrontal cortex; PFCm: medial prefrontal cortex; RH: right hemisphere; SalVentAttnA: salience network type A; SalVentAttnB: salience network type B; SN: salience network.
